# Intestinal protozoan infections shape fecal bacterial microbiota in children from Guinea-Bissau

**DOI:** 10.1371/journal.pntd.0009232

**Published:** 2021-03-03

**Authors:** Sebastian von Huth, Louise B. Thingholm, Poul-Erik Kofoed, Corinna Bang, Malte C. Rühlemann, Andre Franke, Uffe Holmskov

**Affiliations:** 1 Cancer and Inflammation Research, Department of Molecular Medicine, University of Southern Denmark, Denmark; 2 Institute of Clinical Molecular Biology, Christian Albrechts University of Kiel, Germany; 3 Department of Pediatrics, Kolding Hospital, Denmark; 4 Bandim Health Project, INDEPTH Network, Bissau, Guinea-Bissau; University of North Carolina at Chapel Hill, UNITED STATES

## Abstract

Intestinal parasitic infections, caused by helminths and protozoa, are globally distributed and major causes of worldwide morbidity. The gut microbiota may modulate parasite virulence and host response upon infection. The complex interplay between parasites and the gut microbiota is poorly understood, partly due to sampling difficulties in remote areas with high parasite burden. In a large study of children in Guinea-Bissau, we found high prevalence of intestinal parasites. By sequencing of the 16S rRNA genes of fecal samples stored on filter paper from a total of 1,204 children, we demonstrate that the bacterial microbiota is not significantly altered by helminth infections, whereas it is shaped by the presence of both pathogenic and nonpathogenic protozoa, including *Entamoeba* (*E*.) spp. and *Giardia* (*G*.) *lamblia*. Within-sample diversity remains largely unaffected, whereas overall community composition is significantly affected by infection with both nonpathogenic *E*. *coli* (R^2^ = 0.0131, P = 0.0001) and *Endolimax nana* (R^2^ = 0.00902, P = 0.0001), and by pathogenic *E*. *histolytica* (R^2^ = 0.0164, P = 0.0001) and *G*. *lamblia* (R^2^ = 0.00676, P = 0.0001). Infections with multiple parasite species induces more pronounced shifts in microbiota community than mild ones. A total of 31 bacterial genera across all four major bacterial phyla were differentially abundant in protozoan infection as compared to noninfected individuals, including increased abundance of *Prevotella*, *Campylobacter* and two *Clostridium* clades, and decreased abundance of *Collinsella*, *Lactobacillus*, *Ruminococcus*, *Veillonella* and one *Clostridium* clade. In the present study, we demonstrate that the fecal bacterial microbiota is shaped by intestinal parasitic infection, with most pronounced associations for protozoan species. Our results provide insights into the interplay between the microbiota and intestinal parasites, which are valuable to understand infection biology and design further studies aimed at optimizing treatment strategies.

## Author summary

Infections with enteric parasites, including helminths and protozoa, are among the most common infections in poor countries. These infections continue to have enormous impact on both mortality and morbidity worldwide. Enteric parasites inhabit the gut of the infected host, but how infections with these parasites may change the composition of bacteria within the gut remains more or less unclear. Here, we explore how the bacterial composition in stool from children from Guinea-Bissau (Western Africa) is altered by infection with different enteric parasite species. We find that infections with multiple parasite species induces more pronounced changes in the bacterial composition, and that certain parasite species significantly changes the diversity of bacteria. Furthermore, we demonstrate that fecal samples stored at room temperature on filter papers for months can be used in field studies of gut bacteria composition. Our study provides new insights into the complex relationship between enteric parasites and fecal bacteria composition, and may pave the way for new treatment options and help explain why some people are more susceptible to infection compared to others.

## Introduction

Intestinal parasitic infections are among the most common infections in humans and contribute to global morbidity and mortality. Infections are distributed worldwide, with highest prevalence in tropical and subtropical regions, predominantly in developing countries [1]. Intestinal parasitic infections are caused by a wide range of both helminth and protozoan species of varying importance and severity. Soil-transmitted helminths (STHs) include roundworm (*Ascaris lumbricoides*), whipworm (*Trichuris trichiura*) and hookworms (*Necator americanus* and *Ancylostoma duodenale*), and are estimated to affect more than 1.75 billion people worldwide [2,3], and further cause a loss of almost 4 million disability-adjusted life years (DALYs) [4]. Among the most important intestinal protozoans to humans are *G*. *lamblia*, which infects more than 250 million people worldwide [5–8], and *E*. *histolytica*, which contributes with approx. 400,000 cases annually and accounts for 40–110,000 deaths [5,9].

Despite several initiatives to lower incidence, including the distribution of drugs such as metronidazole for *Giardia*-infections and mass drugs for treatment of STHs [10], and improvement of water, sanitation and hygiene [11], intestinal parasitic infections remain a severe global health concern. Although a general decline in STH since 1990 has been documented, a rise in other intestinal parasite infections has been observed, including amebiasis [4], and the infections are still among the most important causes of morbidity in high-prevalent regions. Mass drug treatment campaigns against STHs have highly varying efficacy, especially towards *T*. *trichiura*-infection [12,13]. Additionally, drug resistance is an increasing problem in veterinary medicine, and potentially also in humans, as metronidazole resistance against *E*. spp. and *G*. *lamblia* has been reported [14]. Clearly, there is an urgent need to develop new approaches to control intestinal parasitic infections. One possible way to achieve this is by enhancing understanding of the interaction between intestinal parasites and their host.

The parasite-host relationship is highly complex, and at present only partly understood. It is generally accepted that intestinal parasites have co-evolved with and adapted to their hosts, a feature that is also proposed for prokaryotic microbes of the intestinal tract [15]. To enhance understanding of the interaction between intestinal parasites and their vertebrate host, some focus over the last years has been on how intestinal parasites interacts with the hosts’ gut microbiota, i.e. the commensal microbes (mainly bacteria) within the gastrointestinal tract [15–21]. As the gut microbiota has been demonstrated to be pervasive and essential for human health, including maturation and regulation of the host immune system and protection against pathogens [16–18], and as intestinal parasites share the same niche as the microbiota, it is plausible that a parasite-microbiota-interaction occurs, and that this interaction may determine infection symptomatology, virulence and outcome [19]. Most intestinal parasites secrete immunomodulatory molecules, which may change the local environment within the gut, and thereby induce alterations in the microbiota [20]. Most clinical and preclinical research has focused on how helminths affect the gut microbiota, and the results have not been uniform, as reviewed in [21–23]. The majority of studies undertaken in humans are characterized by a relatively low number of study participants (<100), with variation in both sampling and sequencing technique [21,24]. The microbiota diversity has both been observed to increase and decrease, or even be unaffected due to intestinal helminth infection [24]. For instance, one study reported a decrease in diversity due to infection with *T*. *trichiura* [25], whereas another reported an increase [26]. Further, various bacterial taxa have been associated with infection, but with no clear trend across studies. A recent study covering two geographically separate regions, Liberia and Indonesia, demonstrated that specific members of the gut microbiota could discriminate STH-infected and non-infected individuals. Further, the study demonstrated an increase in *Actinobacteria* spp. due to *T*. *trichiura* infection in a cohort from Liberia, but not in Indonesia [26], which may reflect overall differences in the gut microbiota between geographically separate populations. Additionally, the study found that microbiota diversity was increased in both cohorts due to infection with both *T*. *trichiura*, *A*. *lumbricoides* and hookworms. Similarly, Mejia *et al*. recently found that the fecal microbiota diversity was increased in South American children infected with helminths [27]. In a study conducted in Cameroon, an increased diversity and increase in Bacteroidetes was seen in individuals infected with *Ent*. *histolytica* [28]. This study further highlighted the importance of lifestyle and standard of living on the gut microbiota composition, as the authors studied both hunter-gatherers, farming- and fishing populations. In a study from Côte d’Ivoire, the relative abundance of *Bifidobacteria* and *Escherichia* was demonstrated to increase upon infection with *G*. *lamblia* by targeted qPCR [29], whereas the study by Mejia *et al*. mentioned before found a decreased microbiome diversity in *Giardia*-infected children [27], In a recent study by Berry *et al*., the authors find that *Giardia* infection reduces the abundance of Gammaproteobacteria and increases the abundance of *Prevotella* spp. in more than 1,000 children from four different developing countries [30].

In the present study, we explored associations between alterations in the fecal bacterial microbiota and intestinal parasitic infections in a large cohort of children from the capital of one of the poorest countries in the world, Guinea-Bissau, Western Africa. With this cross-sectional dataset we are unable to infer conclusions on possible pre-existing differences in the microbiota between infected and non-infected subjects. However, based on prior studies as reviewed above that have described the ability of intestinal parasitic infections to induce changes in the gut microbiota, as well as the observed effect of increasing infection load on the microbiota as described below, we present the results with the prerequisite assumption that the observed associations are driven by parasitic infection. From here on the bacterial microbiota is referred to simple as the microbiota. Using this cohort, we have recently investigated the prevalence of intestinal parasitic infections in both healthcare seeking children (referred to as cohort I) and children from the background population (referred to as cohort II), all aged 2–15 years, and found that infections were highly prevalent in both cohorts [31]. In 566 children in cohort I and 708 in cohort II, we found that prevalence of intestinal helminths was 13.8% and 9.6%, respectively (Fisher’s exact test between groups, P = 0.021), whereas prevalence of intestinal protozoa was 41.5% and 46.0%, respectively (Fisher’s exact test between groups, P = 0.112). Helminth infections were mainly due to hookworms, and protozoan infections were dominated by both pathogenic and non-pathogenic species, including *E*. *coli*, *E*. *histolytica*/*dispar* and *G*. *lamblia*. Upon fecal parasitological investigation by microscopy, fecal samples were applied to filter papers, and kept at ambient temperature. By high-quality 16S rRNA gene sequencing (>10,000 reads per sample) of fecal samples from 1,204 of these children, we demonstrate that the fecal microbiota significantly associates with intestinal parasitic infection, and that the association is stronger for children infected with protozoa compared to helminths. Further, we here demonstrate that the fecal microbiota from samples stored at ambient temperature on filter papers (fecal occult blood test, FOBT) for up to 1,000 days can facilitate largescale microbiome studies in remote areas, as is supported by our previous study [32]. We detected a small but significant association of the microbiome with storage time for the current dataset and therefore included storage time as a covariate in further analysis (see S1 Text, S1 and S2 Figs for further details). Thereby, we demonstrate that long-term storage on FOBT papers is an applicable approach for large-scale sample collection in field settings, where immediate freezing of samples is not possible.

To our knowledge, this is the largest study to date investigating the relationship between intestinal parasites and alterations of the fecal microbiota. This exploratory study should enable us to move forward with targeted questions towards understanding the role of the microbiota in intestinal parasitic infections and infection-associated complications.

## Results

### Cohort characteristics and parasite prevalence

The dataset includes microscopic investigation for intestinal parasites in fecal samples from 1,274 children, included between August 2015 and April 2017 in urban Bissau, Guinea-Bissau. Details on the study design, parasitological examination methods and results are described in detail elsewhere [31]. From the cohort, a total of 1,264 fecal samples were applied to FOBT paper, of which 1,253 underwent 16S rRNA sequencing. A total of 60 samples were excluded due to low DNA yield or quality, low sequence reads or recent antibiotic usage, and the final sample size included in this study was 1,204 samples. A flow diagram for the inclusion and final sample size is provided in S3 Fig.

Characteristics of the study participants, as well as storage time of fecal samples on filter paper at room temperature, is provided in Table 1. Of the included subjects with available sequence data, the median age was 6 years in both cohorts (healthcare seeking children and children from the background population) with 54% boys (Table 1). There were no significant differences between the two cohorts with regards to age- and gender distribution. However, there were significant differences in some parameters between the two cohorts, including chicken husbandry, source of drinking water, inclusion period (rainy vs. dry season) and sample storage time (Table 1).

**Table 1:** Cohort characteristics and intestinal parasite prevalence. Characteristics of cohort I (n = 529) and cohort II (n = 675), which were included in the microbiota analysis. Between-group differences are calculated using Wilcoxon rank sum test, Fisher’s exact test or Kruskal–Wallis equality-of-populations rank test, when appropriate.

	Cohort I (n = 529)		Cohort II (n = 675)		Total (n = 1,204)		P
**Gender**							0.367
Male	289	(54.6%)	361	(53.5%)	650	(54.0%)	
Female	240	(45.4%)	314	(46.5%)	554	(46.0%)	
**Age**							0.227
Age, mean (range)	6.7	(2–14)	6.9	(2–15)	6.8	(2–15)	
Age, median	6		6		6		
**Husbandry**							
None	298	(56.3%)	374	(55.4%)	672	(55.8%)	0.397
Pigs	43	(8.1%)	35	(5.2%)	78	(6.5%)	0.181
Ducks	5	(0.9%)	14	(2.1%)	19	(1.6%)	0.247
Chicken	46	(8.7%)	81	(12%)	127	(10.5%)	0.002
Dogs	85	(16.1%)	100	(14.8%)	185	(15.4%)	0.253
Other animals	52	(9.8%)	71	(10.5%)	123	(10.2%)	0.487
**Toilet source**							0.129
Poor	422	(79.8%)	519	(76.9%)	941	(78.2%)	
Good	107	(20.2%)	156	(23.1%)	263	(21.8%)	
**Water source**							<0.001
Poor	319	(60.3%)	171	(25.3%)	490	(40.7%)	
Good	210	(39.7%)	504	(74.7%)	714	(59.3%)	
**Season of inclusion**							0.038
Dry season	255	(48.2%)	366	(54.2%)	621	(51.6%)	
Rainy season	274	(51.8%)	309	(45.8%)	583	(48.4%)	
**Sample storage time at RT**							<0.001
200–300 days	0	(0.0%)	27	(4.0%)	27	(2.2%)	
301–400 days	20	(3.8%)	146	(21.6%)	166	(13.8%)	
401–500 days	83	(15.7%)	124	(18.4%)	207	(17.2%)	
501–600 days	93	(17.6%)	9	(1.3%)	102	(8.5%)	
601–700 days	101	(19.1%)	55	(8.1%)	156	(13.0%)	
701–800 days	63	(11.9%)	94	(13.9%)	157	(13.0%)	
801–900 days	98	(18.5%)	149	(22.1%)	247	(20.5%)	
>900 days	71	(13.4%)	71	(10.5%)	142	(11.8%)	
**Parasite prevalence**							
**Parasite positive (overall)**	**272**	**(51.4%)**	**336**	**(49.8%)**	**608**	**(50.5%)**	**0.817**
Positive for 1 parasite	182	(34.4%)	229	(33.9%)	411	(34.1%)	
Positive for 2 parasites	74	(14.0%)	92	(13.6%)	166	(13.8%)	
Positive for ≥3 parasites	16	(3.0%)	15	(2.2%)	31	(2.6%)	
**Helminth positive**	**74**	**(14.0%)**	**65**	**(9.6%)**	**139**	**(11.5%)**	**0.012**
*Ancylostoma duodenale*	55	(10.4%)	39	(5.8%)	94	(7.8%)	0.002
*Hymenolepis nana*	14	(2.6%)	22	(3.3%)	36	(3.0%)	0.329
**Protozoa positive**	**228**	**(43.1%)**	**306**	**(45.3%)**	**534**	**(44.4%)**	**0.237**
*Entamoeba coli*	50	(9.5%)	48	(7.1%)	98	(8.1%)	0.086
*Entamoeba histolytica/dispar*	95	(18.0%)	119	(17.6%)	214	(17.8%)	0.470
*Giardia lamblia*	112	(21.2%)	173	(25.6%)	285	(23.7%)	0.041
*Endolimax nana*	44	(8.3%)	50	(7.4%)	94	(7.8%)	0.316

Overall parasite prevalence was statistically indifferent between the two cohorts (Table 1): In cohort I, 51.4% were positive for at least one intestinal parasite, whereas 49.8% were positive in cohort II (Fisher’s exact test, P = 0.817). Prevalence of helminths was higher in cohort I than in cohort II (14.0% vs. 9.6%, Fisher’s exact test, P = 0.012), whereas prevalence of protozoa was equal between the two (43.1% vs. 45.3%, Fisher’s exact test, P = 0.237). Prevalence of hookworm and *G*. *lamblia* differed between cohorts (10.4% vs. 5.8%, P = 0.002 and 21.2% vs. 25.6%, P = 0.041, respectively). Infection with multiple species was equally common in the two cohorts; 14.0% of children from cohort I and 13.6% from cohort II were infected with two parasite species, and 3.0% in cohort I and 2.2% in cohort II were infected with three or more parasite species.

The major intestinal helminth species found in the study participants were *Ancylostoma (A*.*) duodenale* (hookworm, n = 94) and *Hymenolepis (H*.*) nana* (dwarf tapeworm, n = 36). The most common intestinal protozoan species found in the participants were *E*. *coli* (n = 98), *E*. *histolytica*/*dispar* (n = 214), *G*. *lamblia* (n = 285) and *Endolimax (E*.*) nana* (n = 94). A number of other intestinal parasite species were found at lower prevalence but were not included in the present study due to lack of statistical power. Prevalence and distribution of the major intestinal parasites in the two cohorts and statistical differences between groups are provided in Table 1.

### Identification of confounding variables

Several host phenotypic and environmental variables have previously been linked to differences in the gut microbiota composition, including age, diet, vitamin supplementation and antibiotic treatment (as reviewed in [33]). Thus, we excluded all individuals with a history of antibiotics use three months prior to inclusion from the analysis, and adjusted for the potential confounding effect of age, history of vitamin A supplementation (binary variable, ((i) yes or (ii) no), toilet source (binary variable, either (i) no private toilet/latrine or (ii) access to private latrine/toilet), tropical season for sample collection (binary variable, (i) rainy or (ii) dry season) and sample storage time (in days). As there were some differences in characteristics and parasite prevalence between the two cohorts (Table 1), all analyses were performed for both cohorts separately, and jointly, the latter adjusted for cohort status. In the following sections, the reported results are from the joined analysis if not stated otherwise.

### Alpha diversity is largely unaffected by intestinal parasite infection

**Fig 1:**
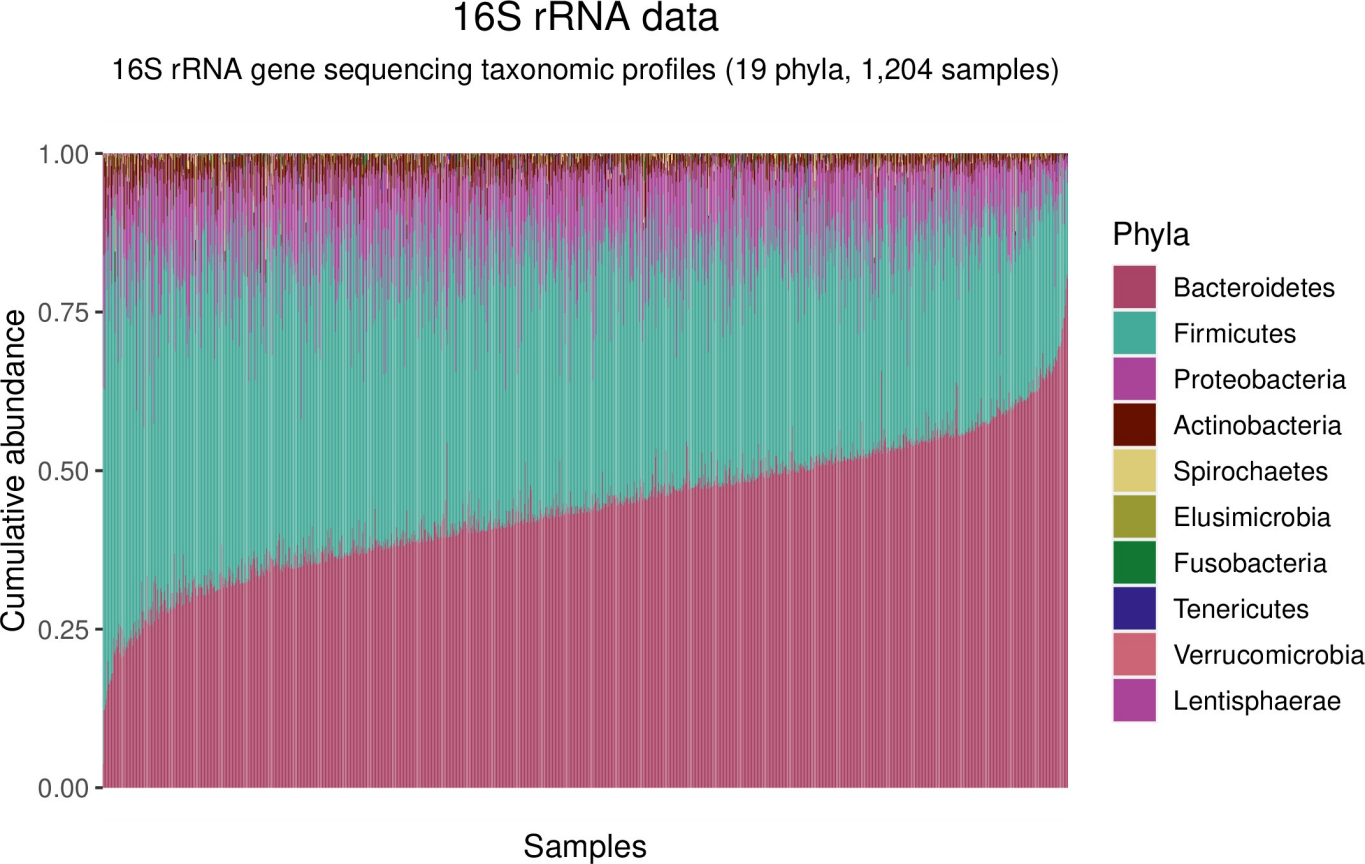
Stacked barplot of taxonomic profile of the gut microbiota across participants. Illustration of the relative abundance (y-axis) of phyla across the 1,204 samples (x-axis) in the study, irrespective of infection status. The 10 most abundant phyla are colored and listed in the legend. For all samples, the two dominant phyla are Bacteroidetes and Firmicutes, followed by Proteobacteria and Actinobacteria, in line with that of a normal human gut microbiota.

Relative abundance of bacterial phyla across all samples revealed that the composition of the microbiota in all study participants was dominated by Firmicutes and Bacteroidetes, as expected (Fig 1).

**Fig 2:**
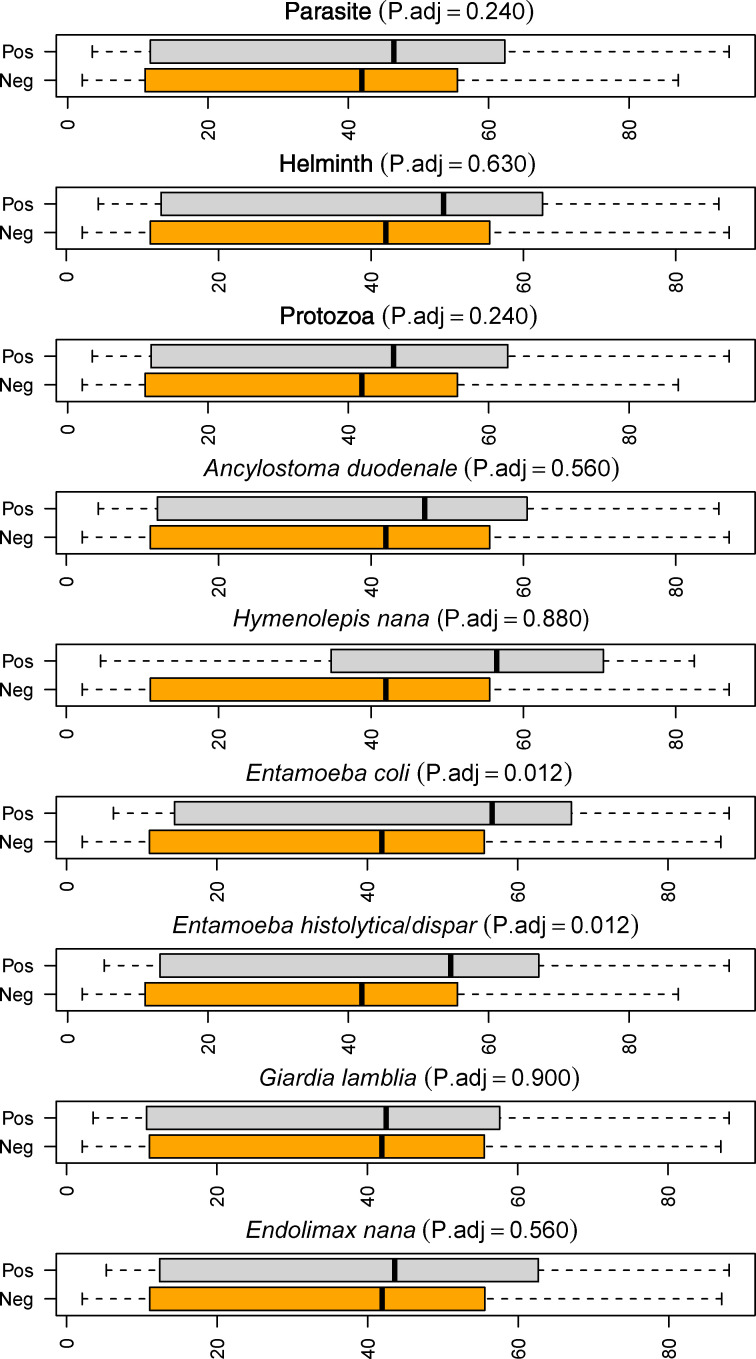
Phylodiversity is altered by infection with *Entamoeba* spp. Illustration of differences in phylodiversity levels between samples with different infection status. Phylodiversity, as a measure of alpha diversity, remains unaltered by infection with helminth species, *G*. *lamblia* and *E*. *nana*. Infection with *E*. spp. significantly increases phylodiversity. Each boxplot shows the non-infected (with any tested parasite (orange) versus samples infected with the parasite of interest as listed in plot title (grey). The adjusted significance level from the robust regression test is given in parentheses.

Three different measures of alpha diversity were calculated to explore possible diversity alterations due to intestinal parasitic infections: Shannon entropy; ACE as a measure of species richness; and phylodiversity as a measure of total unique phylogenetic branch length (Table 2). We compared alpha diversity measurements for individuals with each of nine infection variables (either overall parasite positive, helminth positive, protozoa positive or positive for one of the six specific species, i.e. *A*. *duodenale*, *H*. *nana*, *E*. *coli*, *E*. *histolytica/dispar*, *G*. *lamblia* or *E*. *nana*) against non-infected individuals. Effects on all three diversity indices were limited for all infections, as illustrated in Fig 2 for phylodiversity (Table 2 and Fig 2). A significant decrease in ACE diversity was seen in participants in cohort I with *G*. *lamblia* infection (β = -3.42; P = 0.0470), which was not found in cohort II. Increase in phylodiversity index was seen in cohort II with any intestinal parasite and protozoa (β = 7.00; P = 0.0153 and β = 6.73; P = 0.0266, respectively). Further, all three alpha diversity indices were increased in cohort II with *E*. spp., which were not observed in cohort I. In the pooled dataset containing both cohorts, a significant increase in phylodiversity was seen upon infection with *E*. spp. (β = 11.35; P.adj. = 0.0115 for *Ent*. *coli* and β = 8.15; P.adj. = 0.0115 for *E*. *histolytica/dispar*), however only nominally significant in cohort II. Therefore, changes in alpha diversity was predominantly observed in cohort II, and predominantly for phylodiversity. The changes were mainly an increased diversity in infected individuals in agreement with findings reported in [28].

**Table 2:** Intestinal parasitic infections have limited effects on alpha diversity. Three different alpha diversity indices were analyzed for association with infection status using robust regression (**Methods**). The table show summary statistics: P value for analysis within participants from cohort I, within participants from cohort II and across all participants (Total), Benjamini-Hochberg adjusted P value for analysis across all individuals (P.adj), and association coefficient (Beta). Most pronounced effects are seen in cohort II with protozoan infections. **P values and P.adj values below 0.05 are highlighted**.

	Parasite species	Alpha.div	Cohort I (n = 529)		Cohort II (n = 675)		Total (n = 1,204)		
			Beta	P	Beta	P	Beta	P	P.adj
Overall	Parasite pos. (overall)	PhyloDiv	1.49∙10^00^	6.24∙10^−01^	7.00∙10^00^	1.53∙10^−02^	4.59∙10^00^	3.21∙10^−02^	2.37∙10^−01^
		Shannon	8.52∙10^−04^	9.76∙^10–01^	2.42∙10^−02^	3.65∙10^−01^	1.66∙10^−02^	4.00∙10^−01^	5.84∙10^−01^
		ACE	-8.87∙10^−01^	4.50∙10^−01^	1.44∙10^00^	1.38∙10^−01^	7.00∙10^−01^	3.49∙10^−01^	5.58∙10^−01^
	Helminth pos.	PhyloDiv	2.75∙10^00^	5.79∙10^−01^	3.09∙10^00^	5.89∙10^−01^	2.52∙10^00^	4.92∙10^−01^	6.31∙10^−01^
		Shannon	-1.34∙10^−02^	8.31∙10^−01^	6.59∙10^−02^	3.85∙10^−01^	3.90∙10^−02^	4.11∙10^−01^	5.84∙10^−01^
		ACE	-1.66∙10^−01^	9.47∙10^−01^	-6.28∙10^−01^	8.07∙10^−01^	6.00∙10^−02^	9.74∙10^−01^	9.74∙10^−01^
	Protozoa pos.	PhyloDiv	6.20∙10^−01^	8.61∙10^−01^	6.73∙10^00^	2.66∙10^−02^	3.99∙10^00^	4.41∙10^−02^	2.37∙10^−01^
		Shannon	2.32∙10^−03^	9.42∙10^−01^	2.18∙10^−02^	4.36∙10^−01^	1.37∙10^−02^	5.15∙10^−01^	6.31∙10^−01^
		ACE	-1.34∙10^00^	3.01∙10^−01^	1.70∙10^00^	9.59∙10^−02^	6.17∙10^−01^	4.38∙10^−01^	5.91∙10^−01^
Helminths	*Ancylostoma duodenale*	PhyloDiv	2.46∙10^00^	6.90∙10^−01^	5.37∙10^00^	5.04∙10^−01^	4.29∙10^00^	3.06∙10^−01^	5.58∙10^−01^
		Shannon	-1.96∙10^−03^	9.78∙10^−01^	7.54∙10^−02^	3.75∙10^−01^	5.07∙10^−02^	3.51∙10^−01^	5.58∙10^−01^
		ACE	6.62∙10^−01^	8.13∙10^−01^	-5.82∙10^−01^	8.36∙10^−01^	-2.34∙10^−01^	9.09∙10^−01^	9.44∙10^−01^
	*Hymenolepis nana*	PhyloDiv	8.57∙10^00^	4.98∙10^−01^	-6.08∙10^00^	6.40∙10^−01^	2.80∙10^00^	7.49∙10^−01^	8.79∙10^−01^
		Shannon	-5.60∙10^−02^	6.88∙10^−01^	-1.81∙10^−02^	9.22∙10^−01^	-2.23∙10^−02^	8.38∙10^−01^	9.05∙10^−01^
		ACE	-6.06∙10^00^	2.92∙10^−01^	-1.51∙10^01^	1.64∙10^−01^	-5.69∙10^00^	1.59∙10^−01^	4.77∙10^−01^
Protozoa	*Entamoeba coli*	PhyloDiv	7.14∙10^00^	1.16∙10^−01^	1.64∙10^01^	1.37∙10^−02^	1.13∙10^01^	8.49∙10^−04^	1.15∙10^−02^
		Shannon	7.44∙10^−03^	8.94∙10^−01^	1.37∙10^−01^	2.26∙10^−02^	7.71∙10^−02^	5.93∙10^−02^	2.37∙10^−01^
		ACE	1.20∙10^−01^	9.58∙10^−01^	5.37∙10^00^	7.18∙10^−03^	3.02∙10^00^	4.62∙10^−02^	2.37∙10^−01^
	*Entamoeba histolytica/dispar*	PhyloDiv	6.78∙10^00^	1.29∙10^−01^	9.82∙10^00^	1.42∙10^−02^	8.15∙10^00^	8.38∙10^−04^	1.15∙10^−02^
		Shannon	3.45∙10^−02^	4.36∙10^−01^	7.49∙10^−02^	5.18∙10^−02^	5.47∙10^−02^	6.15∙10^−02^	2.37∙10^−01^
		ACE	-1.13∙10^00^	5.29∙10^−01^	2.91∙10^00^	3.37∙10^−02^	1.36∙10^00^	2.22∙10^−01^	5.08∙10^−01^
	*Giardia lamblia*	PhyloDiv	-2.70∙10^00^	4.87∙10^−01^	2.97∙10^00^	4.20∙10^−01^	5.82∙10^−01^	7.97∙10^−01^	8.97∙10^−01^
		Shannon	-1.77∙10^−02^	6.69∙10^−01^	-4.31∙10^−02^	2.39∙10^−01^	-3.36∙10^−02^	2.18∙10^−01^	5.08∙10^−01^
		ACE	-3.42∙10^00^	4.70∙10^−02^	-2.17∙10^−01^	8.63∙10^−01^	-1.09∙10^00^	2.86∙10^−01^	5.58∙10^−01^
	*Endolimax nana*	PhyloDiv	-3.17∙10^00^	5.30∙10^−01^	8.09∙10^00^	1.53∙10^−01^	3.35∙10^00^	3.28∙10^−01^	5.58∙10^−01^
		Shannon	6.07∙10^−02^	3.21∙10^−01^	7.43∙10^−02^	2.26∙10^−01^	7.42∙10^−02^	8.70∙10^−02^	2.94∙10^−01^
		ACE	-6.56∙10^−01^	7.90∙10^−01^	3.35∙10^00^	8.90∙10^−02^	1.88∙10^00^	2.26∙10^−01^	5.08∙10^−01^

### Highly significant association between protozoa infection and microbiota composition

**Fig 3:**
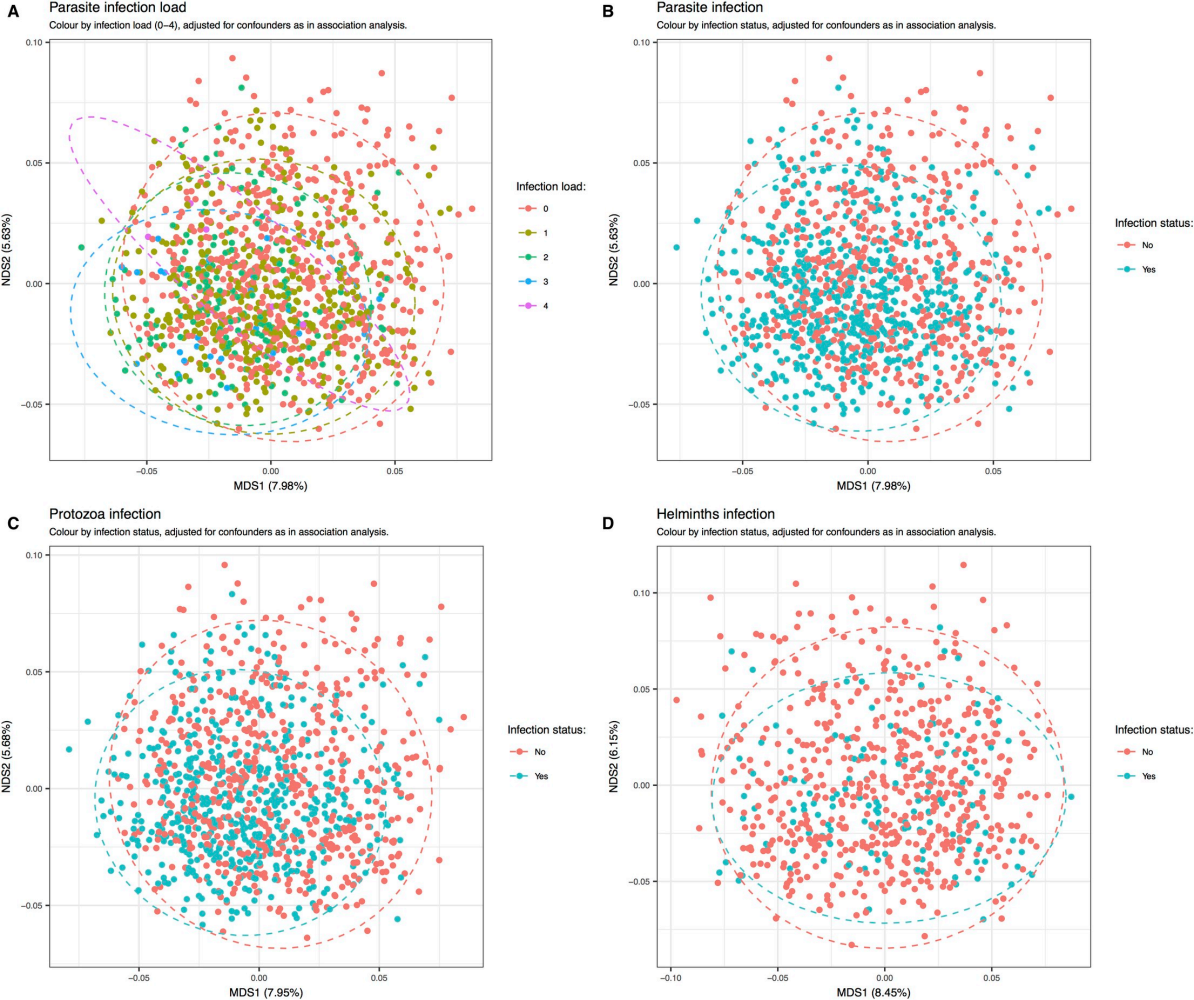
Gut microbiota is shaped by protozoan infections and infection load. **(A)** Increasing infection load, determined by the number of different parasitic species identified by microscopy in each sample, induces increasing shifts in the microbiota, suggesting that multispecies infections have more pronounced effects on microbiota than single species infections. **(B)** Parasite infection (any positive finding, regardless of species) imposes a visual shift in microbiota structure. **(C)** Overall protozoan infection imposes a shift in gut microbiota composition very similar to the shift observed for overall parasite infection, indicating that that protozoa infection, and not helminth infection, has the strongest effect on microbiota composition, an observation supported by results from the adonis-based analysis. **(D)** Helminth infection has no apparent effect on microbiota structure. The figure is composed of four ordination plots build using genera relative abundance data and principal coordinates analysis (capscale function in R package vegan with Bray-Curtis dissimilarity and automatic data transformation (metaMDS = T)). Plots are made with R package ggplot2 and ellipses drawn using function stat_ellipse with default parameters. Each dot shows a sample, all of which are colored by infection status. **(A)** All 1,204 samples colored by infection load from no infection (0) to infected with four different parasite species (4). **(B-D)** Plots showing relationship between the microbiota community of samples either **(B)** non-infected (red) or infected with a parasite (blue), **(C)** non-infected (red) or infected with a protozoa (blue) and **(D)** non-infected (red) or infected with a helminth (blue).

The association between the parasite infection variables and microbial community structure (Bray-Curtis dissimilarity) was evaluated using a permutational multivariate ANOVA-like approach (adonis in R package vegan with 9999 permutations) (Table 3). For overall variables, the most pronounced association was seen for protozoa (R^2^ = 1.03⋅10^−2^; P = 1.00⋅10^−4^) followed by any parasite (R^2^ = 9.73⋅10^−3^; P = 1.00⋅10^−4^) while association for any helminth was insignificant (R^2^ = 2.40⋅10^−3^; P = 6.61⋅10^−2^). Therefore, the association between overall parasite infection and the microbiota may be driven by protozoan infections, as further supported by the similar appearance of the ordination-based visualization of overall parasite and protozoa infections in Fig 3.

**Table 3:** Beta diversity is shaped by protozoa infections. Association between different infection variables and microbiota community structure at genera level, calculated using adonis with 9999 permutations (**Methods**). The most pronounced effects are seen for protozoan infections. Variables marked with red font are significant (P<0.05).

	Parasite variable	R^2^	P
Overall	Parasite pos. (overall)	9.73∙10^−03^	$1.00∙10^{‐04}$
	Helminth pos.	2.40∙10^−03^	6.61∙10^−02^
	Protozoa pos.	1.03∙10^−02^	$1.00∙10^{‐04}$
Helminths	*Ancylostoma duodenale*	1.84∙10^−03^	1.97∙10^−01^
	*Hymenolepis nana*	2.99∙10^−03^	4.62∙10^−02^
Protozoa	*Entamoeba coli*	1.31∙10^−02^	$1.00∙10^{‐04}$
	*Entamoeba histolytica/dispar*	1.64∙10^−02^	$1.00∙10^{‐04}$
	*Giardia lamblia*	6.76∙10^−03^	$1.00∙10^{‐04}$
	*Endolimax nana*	9.02∙10^−03^	$1.00∙10^{‐04}$

Infection load was determined by microscopy and ranked by the number of different parasite species identified in each sample. Most individuals were infected with either none (0) or one (1) parasite species, and with decreasing prevalence two, three or four different species (Table 1). Ordination based evaluation demonstrated that increasing infection load showed an increasing shift in the microbiota community away from the composition of the uninfected individuals (infection load 0), suggesting that multispecies infections induced more pronounced alterations than mild ones (Fig 3), and supported by highly significant association with microbiome composition (adonis, R^2^ = 0.012, P = 0.0001).

Beta diversity was largely unaffected by any helminth variable, whereas the associations for the detected protozoan species (*E*. spp., *G*. *lamblia* and *E*. *nana*) were highly significant. The two *Entamoeba* variables (*E*. *coli* and *E*. *histolytica*/*dispar*) associated with the highest coefficient (R^2^) (R^2^ = 1.31⋅10^−2^; P = 1.00⋅10^−4^ and R^2^ = 1.64⋅10^−2^; P = 1.00⋅10^−4^, respectively). Less pronounced but statistically significant associations were seen in infections with *G*. *lamblia* and *E*. *nana* (R^2^ = 6.76⋅10^−3^; P = 1.00⋅10^−4^ and R^2^ = 9.02⋅10^−3^; P = 1.00⋅10^−4^, respectively) (Table 3).

### A large portion of tested bacterial taxa are associated with intestinal protozoan infection

**Fig 4:**
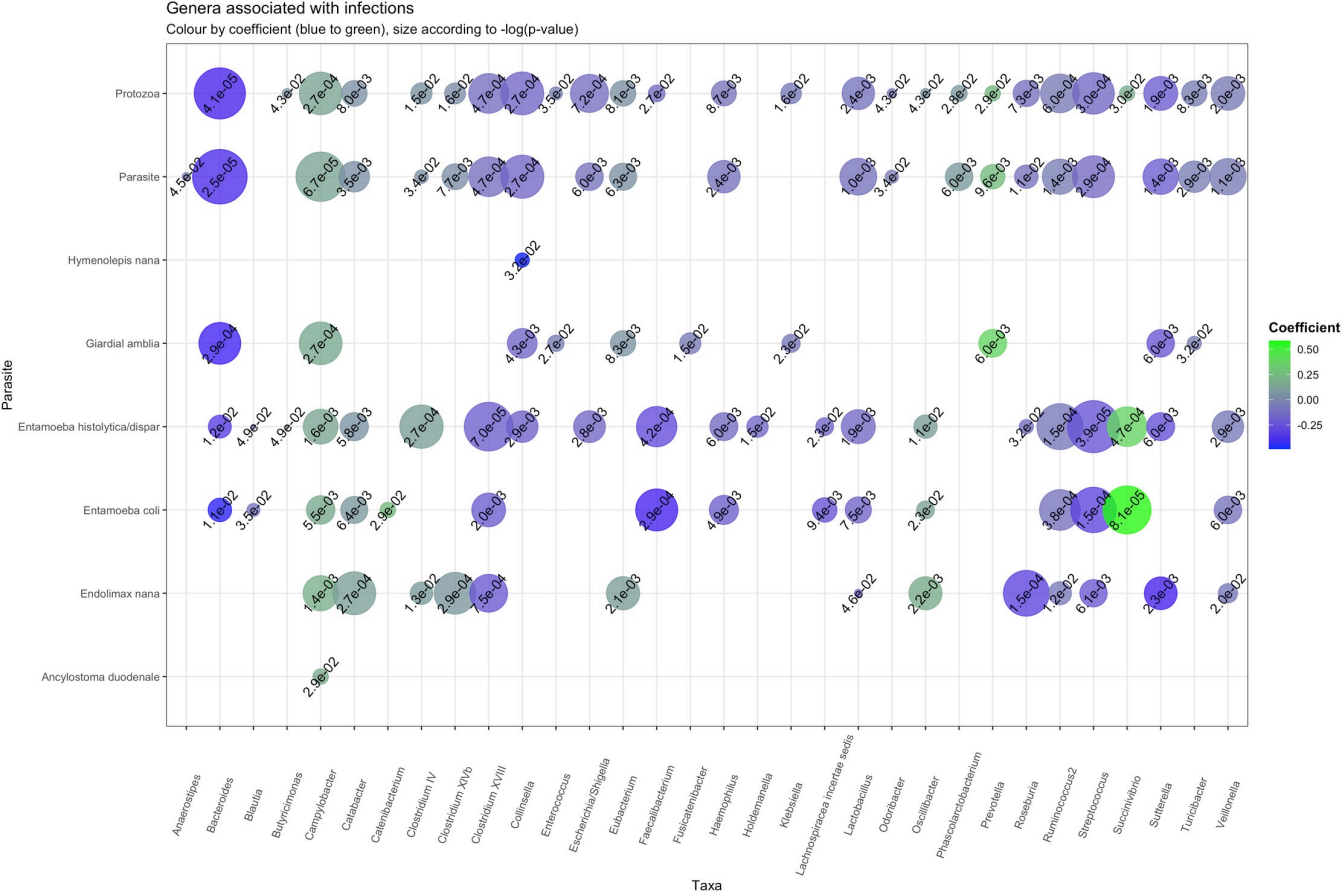
Specific taxa are altered by different parasitic species. A total of 32 genera are significantly associated with intestinal parasitic infection, either overall infections or specific parasite species. Most associations are seen with protozoan infections, whereas helminth infections have limited effects on taxa alterations. The figure illustrates significant associations between genera and different parasite infections (linear regression, P.adj<0.05). Summary statistics for the shown associations are given in Table 4. The color illustrates coefficient (blue for low values, green for high values), whereas size illustrates the significance (calculated by -log(p-value) (the larger bubble, the higher significance). The plot is made using ggplot2 package in R.

Associations between the parasite infection variables and the relative abundance of individual taxa was evaluated for the phylogenetic level phylum to genus (Table 4 and Fig 4). As mentioned, only minor effects on the fecal microbiota beta-diversity were observed in individuals with helminth infections (Fig 3). Accordingly, we observed only few associations regarding specific taxa of the fecal microbiota and helminth infections; for the overall helminth variable no associations remained significant at genus level (P.adj.<0.05) while the Epsilonproteobacteria-Campylobacterales-Campylobacteraceae branch was increased in abundance for individuals infected with *A*. *duodenale* (Table 4). However, *Campylobacter* abundance was broadly increased across individuals infected with protozoa, thereby causing the association with *A*. *duodenale* to be non-specific (Fig 4 and Table 4). Beyond *Campylobacter*, the only genus associating with helminth infection was *Collinsella* that associated with *H*. *nana* infection (β = -0.48; P.adj. = 3.22⋅10^−2^).

**Table 4:** Alterations of individual genera due to intestinal protozoa infections. A total of 31 genera from the four major phyla of the gut microbiota were either found to have significantly increased or decreased abundance due to protozoan infections (linear regression, P.adj< 0.05). The *Campylobacter* genus was found at increased abundance due to any of the protozoan infections. The table shows coefficients (Beta) and Benjamini-Hochberg adjusted P values (P.adj) for each association with P.adj<0.05. The associations were evaluated for genera and the higher taxonomic levels are listed to support evaluation of affected phylogenetic branches.

Taxa					Protozoa		*Ent*. *coli*		*Ent*. *histolytica/dispar*		*Giardia lamblia*		*Endolimax nana*	
Phylum	Class	Order	Family	Genera	Beta	P.adj	Beta	P.adj	Beta	P.adj	Beta	P.adj	Beta	P.adj
Actinobacteria	Coriobacteriia	Coriobacteriales	Coriobacteriaceae	*Collinsella*	-1.63∙10^−01^	2.66∙10^−04^			-1.82∙10^−01^	2.91∙10^−03^	-1.63∙10^−01^	4.32∙10^−03^		
Bacteroidetes	Bacteroidia	Bacteroidales	Bacteroidaceae	*Bacteroides*	-3.89∙10^−01^	4.12∙10^−05^	-4.62∙10^−01^	1.11∙10^−02^	-3.37∙10^−01^	1.18∙10^−02^	-4.23∙10^−01^	2.87∙10^−04^		
Bacteroidetes	Bacteroidia	Bacteroidales	Odoribacteraceae	*Butyricimos*	5.03∙10^−02^	4.28∙10^−02^			6.65∙10^−02^	4.90∙10^−02^				
Bacteroidetes	Bacteroidia	Bacteroidales	Odoribacteraceae	*Odoribacter*	-5.32∙10^−02^	4.28∙10^−02^								
Bacteroidetes	Bacteroidia	Bacteroidales	Prevotellaceae	*Prevotella*	2.40∙10^−01^	2.93∙10^−02^					3.80∙10^−01^	6.00∙10^−03^		
Firmicutes	Bacilli	Lactobacillales	Enterococcaceae	*Enterococcus*	-3.31∙10^−02^	3.45∙10^−02^					-4.37∙10^−02^	2.74∙10^−02^		
Firmicutes	Bacilli	Lactobacillales	Lactobacillaceae	*Lactobacillus*	-9.95∙10^−02^	2.42∙10^−03^	-1.75∙10^−01^	7.52∙10^−03^	-1.44∙10^−01^	1.92∙10^−03^			-1.39∙10^−01^	4.57∙10^−02^
Firmicutes	Bacilli	Lactobacillales	Streptococcaceae	*Streptococcus*	-1.48∙10^−01^	2.97∙10^−04^	-3.05∙10^−01^	1.52∙10^−04^	-2.50∙10^−01^	3.92∙10^−05^			-2.27∙10^−01^	6.07∙10^−03^
Firmicutes	Clostridia	Clostridiales	Lachnospiraceae	*Blautia*			-1.01∙10^−01^	3.45∙10^−02^	-7.01∙10^−02^	4.90∙10^−02^				
Firmicutes	Clostridia	Clostridiales	Catabacteriaceae	*Catabacter*	4.46∙10^−02^	8.04∙10^−03^	8.39∙10^−02^	6.36∙10^−03^	6.28∙10^−02^	5.80∙10^−03^			1.22∙10^−01^	2.66∙10^−04^
Firmicutes	Clostridia	Clostridiales	Clostridiaceae	*Clostridium_IV*	5.32∙10^−02^	1.51∙10^−02^			1.14∙10^−01^	2.66∙10^−04^			1.02∙10^−01^	1.26∙10^−02^
Firmicutes	Clostridia	Clostridiales	Clostridiaceae	*Clostridium_XlVb*	2.90∙10^−02^	1.55∙10^−02^							8.30∙10^−02^	2.87∙10^−04^
Firmicutes	Clostridia	Clostridiales	Clostridiaceae	*Clostridium_XVIII*	-1.22∙10^−01^	4.71∙10^−04^	-2.07∙10^−01^	1.96∙10^−03^	-1.98∙10^−01^	6.97∙10^−05^			-2.25∙10^−01^	7.52∙10^−04^
Firmicutes	Clostridia	Clostridiales	Eubacteriaceae	*Eubacterium*	4.82∙10^−02^	8.10∙10^−03^					6.01∙10^−02^	8.32∙10^−03^	1.05∙10^−01^	2.13∙10^−03^
Firmicutes	Clostridia	Clostridiales	Ruminococcaceae	*Faecalibacterium*	-1.32∙10^−01^	2.66∙10^−02^	-4.02∙10^−01^	2.87∙10^−04^	-2.82∙10^−01^	4.15∙10^−04^				
Firmicutes	Clostridia	Clostridiales	Lachnospiraceae	*Fusicatenibacter*							-5.96∙10^−02^	1.51∙10^−02^		
Firmicutes	Clostridia	Clostridiales	Oscillospiraceae	*Oscillibacter*	6.34∙10^−02^	4.28∙10^−02^	1.32∙10^−01^	2.35∙10^−02^	1.08∙10^−01^	1.11∙10^−02^			1.82∙10^−01^	2.18∙10^−03^
Firmicutes	Clostridia	Clostridiales	Lachnospiraceae	*Roseburia*	-1.11∙10^−01^	7.32∙10^−03^			-1.27∙10^−01^	3.22∙10^−02^			-3.10∙10^−01^	1.48∙10^−04^
Firmicutes	Clostridia	Clostridiales	Ruminococcaceae	*Ruminococcus2*	-5.35∙10^−02^	6.00∙10^−04^	-1.05∙10^−01^	3.76∙10^−04^	-8.43∙10^−02^	1.48∙10^−04^			-7.70∙10^−02^	1.16∙10^−02^
Firmicutes	Erysipelotrichia	Erysipelotrichales	Erysipelotrichaceae	*Catenibacterium*			2.50∙10^−01^	2.93∙10^−02^						
Firmicutes	Erysipelotrichia	Erysipelotrichales	Erysipelotrichaceae	*Holdemanella*					-1.55∙10^−01^	1.49∙10^−02^				
Firmicutes	Erysipelotrichia	Erysipelotrichales	Erysipelotrichaceae	*Turicibacter*	-3.90∙10^−02^	8.32∙10^−03^					-4.25∙10^−02^	3.24∙10^−02^		
Firmicutes	Negativicutes	Acidaminococcales	Acidaminococcaceae	*Phascolarctobacterium*	7.92∙10^−02^	2.77∙10^−02^								
Firmicutes	Negativicutes	Veillonellales	Veillonellaceae	*Veillonella*	-5.49∙10^−02^	1.98∙10^−03^	-9.43∙10^−02^	6.01∙10^−03^	-7.40∙10^−02^	2.91∙10^−03^			-8.27∙10^−02^	1.98∙10^−02^
Proteobacteria	Betaproteobacteria	Burkholderiales	Sutterellaceae	*Sutterella*	-2.10∙10^−01^	1.92∙10^−03^			-2.62∙10^−01^	6.00∙10^−03^	-2.32∙10^−01^	6.01∙10^−03^	-3.95∙10^−01^	2.32∙10^−03^
Proteobacteria	Gammaproteobacteria	Aeromonadales	Succinivibrionaceae	*Succinivibrio*	1.77∙10^−01^	3.02∙10^−02^	5.85∙10^−01^	8.10∙10^−05^	3.79∙10^−01^	4.71∙10^−04^				
Proteobacteria	Gammaproteobacteria	Enterobacterales	Enterobacteriaceae	*Escherichia*.*Shigella*	-1.24∙10^−01^	7.20∙10^−04^			-1.53∙10^−01^	2.81∙10^−03^				
Proteobacteria	Gammaproteobacteria	Enterobacterales	Enterobacteriaceae	*Klebsiella*	-6.19∙10^−02^	1.61∙10^−02^					-7.51∙10^−02^	2.27∙10^−02^		
Proteobacteria	Gammaproteobacteria	Pasteurellales	Pasteurellaceae	*Haemophilus*	-9.47∙10^−02^	8.67∙10^−03^	-1.91∙10^−01^	4.89∙10^−03^	-1.36∙10^−01^	6.00∙10^−03^				
Proteobacteria	Epsilonproteobacteria	Campylobacterales	Campylobacteraceae	*Campylobacter*	1.38∙10^−01^	2.66∙10^−04^	1.68∙10^−01^	5.52∙10^−03^	1.49∙10^−01^	1.58∙10^−03^	1.66∙10^−01^	2.66∙10^−04^	1.98∙10^−01^	1.45∙10^−03^
				*Lachnospiracea incertae sedis*			-2.45∙10^−01^	9.43∙10^−03^	-1.64∙10^−01^	2.35∙10^−02^				

For the 43 genera analyzed, a total of 31 genera from the four major gut microbiota phyla associated with protozoa infection, either overall protozoa positive, or for individual species (P.adj.<0.05) (Table 4 and Fig 4). Most genera were found to be altered by overall protozoa infection (10 with increased abundance, 16 with decreased abundance), followed by infection with *E*. *histolytica/dispar* (6 with increased abundance, 15 with decreased abundance), *E*. *coli* (5 with increased abundance, 10 with decreased abundance), *E*. *nana* (6 with increased abundance, 7 with decreased abundance) and *G*. *lamblia* (3 with increased abundance, 7 with decreased abundance) (Table 4 and Fig 4).

The genus *Collinsella* within the Actinobacteria phylum was associated with both *E*. *histolytica/dispar* and *G*. *lamblia* infection (β = -1.82⋅10^−1^; P.adj. = 2.91⋅10^−3^ and β = -1.63⋅10^−1^; P.adj. = 4.32⋅10^−3^, respectively). Within the Bacteroidetes phylum, the genus *Prevotella* was associated with *G*. *lamblia* infection (β = 3.80⋅10^−1^; P.adj. = 6.00⋅10^−3^, Table 4 and Fig 4).

Nineteen genera within the Firmicutes phylum were associated with protozoa infections. Overall protozoa infection was associated with lower abundance of genera within the Lactobacillales order, including *Enterococcus* (β = -3.31⋅10^−2^; P.adj. = 3.45⋅10^−2^), *Lactobacillus* (β = -9.95⋅10^−2^; P.adj. = 2.42⋅10^−3^) and *Streptococcus* (β = -1.48⋅10^−1^; P.adj. = 2.97⋅10^−4^), and these trends were found for specific protozoa species as well (Table 4 and Fig 4). Genera within the Clostridiales order were found associated with both increased and decreased abundance, depending on protozoan species. For example, decreased abundance of *Blautia* was observed in individuals with *E*. *coli* (β = -1.01⋅10^−1^ P.adj. = 3.45⋅10^−2^) and *E*. *histlytica*/*dispar* (β = -7.01⋅10^−2^; P.adj. = 4.90⋅10^−2^), whereas abundance of the genus *Clostridium IV* was increased by *E*. *histolytica*/*dispar* and *E*. *nana*, but not by *E*. *coli* or *G*. *lamblia* (Table 4 and Fig 4). Decreased abundance of the closely related genus *Clostridium* XVIII was associated with all protozoa parasites except *G*. *lamblia* (Table 4 and Fig 4).

Of the four tested genera within the Gammaproteobacteria class, three were found to be less abundant in individuals with protozoan infections, namely *Escherichia/Shigella*, *Klebsiella* and *Haemophilus*, while the fourth, *Succinivibrio*, was increased (Table 4 and Fig 4).

One genus within the Epsilonproteobacteria class, namely *Campylobacter*, was the only genus found with increased abundance for all of the aforementioned protozoan infection variables (β = 1,38⋅10^−1^; P = 2,66⋅10^−4^ for overall protozoa infection, β = 1.68⋅10^−1^; P.adj. = 5.52⋅10^−3^ for *E*. *coli* infection, β = 1.49⋅10^−1^; P.adj. = 1.58⋅10^−3^ for *E*. *histolytica/dispar* infection, β = 1.66⋅10^−1^; P.adj. = 2.66⋅10^−4^ for *G*. *lamblia* infection, β = 1.98⋅10^−1^; P.adj. = 1.45⋅10^−3^ for *E*. *nana* infection) (Table 4 and Fig 4).

Additionally, the *Lachnospiracea incertae sedis* family (at present taxonomically organized within the Clostridia class) was found at lower abundance by infection with any *E*. spp. (β = -2.45⋅10^−1^; P.adj. = 9.43⋅10^−3^ for *E*. *coli* and β = -1.64⋅10^−1^; P.adj. = 2,35⋅10^−2^ for *E*. *histolytica/dispar*) (Table 4 and Fig 4).

In summary, a large portion of the analyzed genera associated with protozoa infection, while only few taxa were significantly associated with helminth infection. All major phyla normally found in the human gut microbiota were represented across associated genera.

## Discussion

In the present study, we analyzed fecal microbiota composition from 1,204 children from Bissau, Guinea-Bissau, with an overall high prevalence of intestinal parasitic infections, predominantly caused by protozoans including *E*. spp. and *G*. *lamblia*. We demonstrated that microbial alpha diversity was largely unaffected by helminth infections, and that protozoan infections had moderate effects on alpha diversity. We showed that beta diversity associated with infection status for both pathogenic and non-pathogenic protozoa, and that the abundance of a total of 32 bacterial genera were altered due to parasite infections. Finally, we demonstrated the value of FOBT papers for microbiota sampling in rural areas.

We found a total of 31 genera from four different phyla, out of 43 genera analyzed, to significantly associate with intestinal protozoan infection. These include a decrease of the *Collinsella* genus in individuals infected with *E*. *histolytica*/*dispar* and *G*. *lamblia*. To our knowledge, no previous studies have associated this genus with intestinal parasitic infections, however, the Actinobacteria phylum has recently been demonstrated to be increased upon infection with *Trichuris trichiura* in humans [26]. *Collinsella* spp. have been demonstrated to regulate levels of circulating insulin in pregnant women [34], and a reduced abundance has been associated with symptom severity in patients with irritable bowel syndrome [35]. A common and debilitating feature of *Giardia* infection is the post-giardiasis syndrome after complete elimination of the parasite, with a symptomatology very much alike irritable bowel syndrome [36,37]. One possible explanation for these long-lasting post-infectious symptoms could be an altered microbiota, including decreased abundance of *Collinsella* spp. However, as *G*. *lamblia* resides in the small intestine only [38], an effect seen in the fecal microbiota may be immunological derived, rather than by local interactions. In mice, *Giardia* infection has been reported to increase the abundance of Proteobacteria in the fore- and hindgut [39]. This is contradictory to our observations, where Proteobacteria itself was not associated and two of three genera in the clade were decreased upon *Giardia* infection.

We found a decreased abundance of the *Bacteroides* genus due to infection with *E*. *histolytica/dispar*. There are conflicting results regarding gut microbiota alterations due to *E*. *histolytica* infection, as both increased [28] and decreased [40] abundance of *Bacteroides* has previously been observed. The two studies in question are conducted in Zimbabwe and India, respectively, and the conflicting findings could be due to geographical changes in gut microbiota. Further, there are differences in the applied technique, as one is based on sequencing, and the other on targeted PCR. We further found a decrease in *Lactobacillus* spp. due to infection with *E*. *histolytica/dispar*, which is consistent with previous findings [40].

We found limited (single taxa and beta-diversity) or no (alpha-diversity) effects on gut microbiota composition due to helminth infections. This is contradictory to previous studies on the subject, in which several bacterial taxa have been associated with infection [41]. Regarding hookworm infection, previous studies have demonstrated an increase in Bacteroidetes and a decrease in both *Lachnospiraceae* and Firmicutes [26,42]. The number of hookworm-infected individuals in these studies vary between 8 and 55, compared to a total of 94 in the present study, and the differences could thus be explained by a lack of power in the previous studies, as these hold an increased risk of false positive findings. Furthermore, helminth prevalence is minor in the present study, compared to protozoa, and may partly explain why less pronounced effects due to helminth infections are observed. Further, regional and geographical differences in gut microbiota composition is another plausible explanation for the lack of uniform results. To our knowledge, no other studies have investigated gut microbiota alterations due to infection with *Hym*. *nana*.

Historically, all protozoa and helminths were considered parasitic, and assumed to be pathogenic. As reflected by the number of prevalent cases and related morbidity worldwide, this is indeed true for some species. A distinctive feature of many intestinal parasitic infections is that they cause significant morbidity, and less pronounced mortality. For instance, STH infections, which especially affect children, may cause nutritional deficiency, which may lead to anemia and ultimately reduced growth and cognitive development [2,3,43]. With regards to some intestinal parasites, infection can be life-threatening and even fatal, as seen for hyperinfection syndrome of *Strongyloides* infection, bowel obstruction in *Ascaris* infection, or invasive amebiasis by *E*. *histolytica* infection [44–46]. Although some intestinal parasites may cause pronounced pathology in humans, evaluation of the existing literature indicates that many common eukaryotic species within the human gut, originally identified as pathogenic parasites, are actually commensals or even beneficial, at least in part, and could be regarded as pathobionts, only causing disease in certain contexts [23,47,48]. Some even extend this to state that eukaryotic members of the microbiota (termed the eukaryome or parasitome) are crucial in maintaining gut homeostasis and shaping host immunity [49], and that consequently, absence of e.g. helminths may result in a dysfunctional immune system [15], which partially explain the rise in autoimmune diseases seen in the industrialized world [50]. The most prevalent intestinal parasites found in the present study are pathogenic, with the exception of the amebic species *E*. *coli* and *E*. *nana*, which are generally accepted as being non-pathogenic. We demonstrate that there are only minor differences between the two cohorts (children seeking medical attention and children found in the background population, respectively) with regard to parasite prevalence, and this difference is due to helminth infections, which are dominant in cohort I. By so, our findings support that the presence of intestinal parasites does not necessarily cause individuals to seek medical attention, even though the microbiota of these individuals is altered.

One major limitation of our study is the traditional and rather rough method for detecting intestinal parasites. In the industrialized world, conventional light microscopy has largely been replaced by molecular diagnostics including qPCR, which has proven to be superior to microscopy with increased sensitivity and specificity [51]. However, in developing countries and field settings, laboratory access is sparse, and microscopy remains a cheap, fast and reproducible method for parasite examination, with acceptable sensitivity and specificity [52]. With the lower sensitivity of microscopy as compared to qPCR methods, there is a risk that parasite infections have gone undetected in the current study. While the relatively insensitive light microscopy might ensure that the parasite burden in positive samples is clinically relevant, it entails a risk that sub-clinical and undetected infections could still skew the interpretations. Another methodological limitation with microscopy diagnosis is the failure to differentiate between pathogenic and potentially fatal *E*. *histolytica* and non-pathogenic *E*. *dispar*, as the two are indistinguishable by microscopy [53,54]. The high prevalence of *E*. *histolytica/dispar* found in our study may very likely resemble *E*. *dispar*, and the described associations with fecal microbiota may not be due to *E*. *histolytica*. Microbiota alterations due to infection with *E*. *histolytica* has previously been investigated in Cameroon, where an infection-dependent increase in Bacteroidetes was reported [28].

Due to the sequencing approach used to investigate the fecal microbiota, we were unable to detect alterations on strain-level. Furthermore, as the two genera *Escherichia* and *Shigella* (within the Gammaproteobacteria class) have very similar 16S rRNA gene sequences, we were unable to differentiate between the two. Both genera are related to gastrointestinal pathology, and differentiation between the two by other approaches would possibly yield interesting aspects. Finally, the use of samples stored on FOBT paper at room temperature could be considered as a weakness of the present study. Although we demonstrated some significant alterations due to room temperature storage and accordingly adjusted for storage time, such changes were minor, and do not argue against the use of FOBT cards in fieldwork without electricity, as we also demonstrated in a previous study [32]. What seems to be important is a uniform sample collection, and that analyses are adjusted for storage time.

In the present study, we demonstrate that microbiota assemblages are significantly associated with intestinal protozoa infections, whereas limited or no effects are seen due to helminth infections. We find that specific taxa associated with different protozoan infections, which can form the basis for further targeted analysis with the aim of developing approaches that improve treatment, symptom relief or colonization resistance through modulating the microbiome. For now, this study further enhances our understanding of the interplay between host microbiota and intestinal parasitic infections.

## Materials and methods

### Ethical statement

The participant enrollment and microscopic investigation of fecal samples was approved by the Ethical Committee of Guinea-Bissau (Comité Nacional de Ética na Saúde) (ref. no. 0029/CNES/INASA/2015). Participants or parents/guardians to participants gave oral and written consent to participate. Subsequent microbiota analysis was approved by the Ethical Committee of Guinea-Bissau (ref. no. 062/CNES/INASA/2017) and the Regional ethics committee of Region of Southern Denmark (ref. no. S-20160138). The study was conducted in adherence to the Declaration of Helsinki.

### Sample collection and storage

Stool samples were collected as a part of a prospective no-intervention two-cohort study, investigating the prevalence and potential risk factors for intestinal parasite infections in children from Bissau, Guinea-Bissau, Western Africa. The study area and sample collection procedure has been described in detail previously [31]. In brief, children aged 2–15 years were included between August 2015 and April 2017 at local health centers (cohort I) or at their private address (cohort II). Upon inclusion, participants delivered fresh stool samples in designated sterile containers, which were kept in a refrigerator prior to microscopic analysis for intestinal parasites. Microscopic parasitological analyses were performed following the local routine, and infection load was determined by the number of different species identified. Upon microscopic investigation, the fecal sample was manually homogenized within the container, and approximately 0.5 mL of the sample was applied to the two filter paper windows of a fecal occult blood test (FOBT) filter card (Hemoccult, Beckman Coulter) with a clean wooden spatula. The sample was air-dried under laminar airflow, protected from sunlight, for 1–6 hours, after which the sample was packed in an individual airtight zip lock bag with desiccant (Whatman desiccant packs, Sigma-Aldrich). Samples were subsequently stored in the dark at ambient temperature, which is approx. 25°C on average in Guinea-Bissau [55], prior to airplane shipment to laboratory facilities in Germany for DNA extraction and 16S rRNA sequencing. Sample storage time was calculated from day of inclusion to the day of DNA extraction. All samples were stored between 209 and 993 days at room temperature prior to DNA extraction.

The storage on filter paper was chosen due to lack of freezing capacity and further lack of a possibility to transport samples from Guinea-Bissau to central laboratory facilities at stable and constant freezing temperatures. We have recently demonstrated that this particular storage method is applicable in microbiota research, as the fecal microbiota from samples stored on FOBT filter papers at room temperature for up to five months is comparable to that of a sample frozen and kept at -80°C immediately after collection, with regards to diversity and cumulative abundances [32].

A total of 1,274 fecal samples were collected for microscopic investigation, all from participants with complete questionnaire data. Of these, samples from 1,264 participants were applied to filter paper and underwent DNA extraction as described below. A flow diagram of the study is provided in S3 Fig.

### DNA extraction

The actual filter from the FOBT cards was cut free from the card using scissors and handled using tweezers. Instruments were cleansed thoroughly between each sample using absolute ethanol (EMSURE) to avoid cross-contamination between samples. Bacterial DNA extraction from FOBT papers was performed with the QIAamp DNA Stool Mini Kit (QIAGEN) on a QIAcube platform (QIAGEN), according to manufacturer’s instructions with minor modifications. In brief, the FOBT paper was placed in PowerBead Tubes with Garnet beads (0.70 mm) (QIAGEN) with ASL lysis buffer (QIAGEN). Samples were homogenized by bead beating at 40–50 MHz for 45 seconds on a SpeedMill PLUS instrument (Analytik Jena AG), spun down and the supernatant stabilized with InhibitEX tablets (QIAGEN), containing PCR inhibitor absorption matrix. Subsequent DNA extraction was automated on the QIAcube, following standard programs. Extracted DNA was stored at -80°C prior to PCR amplification. Blank non-template extraction controls were included to investigate potential contamination.

### Bacterial DNA amplification and pooling

The two hypervariable regions V1 and V2 of the 16S rRNA gene were amplified using the forward 27F primer and reverse 338R primers and dual MID indexing, as described by Kozich *et al*. [56]. Bacterial DNA was dually barcoded by unique forward and reverse primers, as described by Caporaso *et al*. [57], enabling subsequent multiplexing of the PCR product. PCR products were evaluated by gel analysis and normalized using the SequalPrep Normalization plate Kit (Invitrogen), according to manufacturer’s instructions. The pooled PCR products were measured fluorometrically using Qubit 4 Fluorometer (Invitrogen), to test DNA concentration.

### 16S rRNA gene sequencing and data processing

Sequencing was performed on the Illumina MiSeq platform, using the MiSeq Reagent Kit v3 according to manufacturer’s instructions. MiSeq FastQ files were trimmed using sickle [58] in PE (paired-end) mode with a sliding window of 0.1 readlength. Trimming was performed when average quality within the window was below 20, and reads were all >100 bp after trimming. Reads were stitched using VSEARCH [59] with a length between 280 and 350 bp. Further, VSEARCH filtered reads with more than 1 expected error. Further quality filtering was performed using the FastX-Toolkit::fastq_quality_filter [60] to exclude sequences with >5% nucleotides with a quality score below 30. Files were subsequently converted to FASTA format, and chimeras were removed in VSEARCH, using the gold.fa database. The remaining reads were classified using the UTAX algorithm, where reads classified as chloroplasts or not classified at domain level were removed.

OTU tables were generated in UPARSE [61], implemented in VSEARCH. After removal of replicates and singletons, reads were clustered based on 97% similarity. Chimeras were once again filtered using VSEARCH in de-novo mode. To generate OTU abundance tables, all reads per sample were mapped to OTU tables using VSEARCH. Using the SINTAX classifier [62] at lowest possible level with minimum 80% bootstrap confidence, one representative sequence for each OTU was annotated. OTUs with identical annotations were grouped into taxonomic bins. Samples from individuals with self-reported antibiotic usage 3 months prior to inclusion were not included in the analysis (n = 31).

### Statistical analysis

Between-group differences in baseline characteristics and parasite prevalence were calculated using Wilcoxon rank-sum test, Fisher’s exact test and Kruskal-Wallis rank test in STATA 15.1 (StataCorp, College Station, TX, USA). P-values <0.05 were considered significant in these analyses.

Statistical analysis of the microbiota data was performed using the R programming environment v3.2 [63]. All adjusted p-values were obtained using the P.adjust function in R package stats and the Benjamini-Hochberg method (method = “BH”). First, data was filtered by excluding samples with less than 10,000 reads and samples possibly affected by overgrowth of facultative anaerobic taxa, which were predominantly found in the Proteobacteria and Firmicutes phyla (the latter in the branch of *Streptococci*). These samples were identified if they fell above the third quartile plus three times inter quantile range (IQR) of phylum abundance (n = 18). Microbial count data on the remaining 1,204 samples was transformed to adjust for deviating sequencing depth by dividing the counts by sample sum and multiplied by one hundred to obtain relative abundances between zero and 100.

As the life cycle, infection route and severity of infection vary considerably between different species of intestinal parasites, we chose to analyze different aspects of the fecal microbiota separately. First, we performed analyses for any parasite, any helminth and any protozoa, and subsequently performed analysis for the most abundant individual pathogenic parasite species (*A*. *duodenale*, *H*. *nana*, *E*. *coli*, *E*. *histolytica/dispar*, *G*. *lamblia* and *E*. *nana*). For each analysis, the infected group was compared with the subset of individuals with no detectable parasitic infection (n = 596).

All analyses were adjusted for storage time at room temperature. Alterations associated with helminth infections were adjusted for co-infection with protozoa, and vice versa. To further control for potential confounding factors, all analyses were adjusted for age, usage of vitamin A, toilet source and tropical season of sample collection. As described above, participants were enrolled in the study on a two-cohort basis. While there was no overall difference in infection load between the two cohorts, prevalence of some species differed significantly, and we thus adjusted for cohort status in the joint analysis and performed a separate supportive analysis within each group of the two cohorts. Results from the joined analysis is provided in the main text while S1 Table show results from all three analyses.

### Association with alpha- and beta-diversity

Associations between alpha-diversity and each of the nine infection states (overall parasite positive; overall helminth positive; overall protozoa positive; *A*. *duodenale* positive; *H*. *nana* positive, *E*. *coli* positive, *E*. *histolytica/dispar* positive, *G*. *lamblia* positive, *E*. *nana* positive) was evaluated using a robust regression (lmRob function in R package robust [64]) and the covariates given above. The alpha diversity measures considered were Shannon entropy (diversity function with index = “shannon” in R package vegan [65]), the measure of species richness ACE (estimateR function using result row four in R package vegan), and phylodiversity as a measure of total unique phylogenetic branch length (calculated using mothur’s phylo.diversity function [66] with the phylogenetic tree built using FastTree with—nt and—gtr and the 16S OTU table as input). Evaluation of the association between the parasite infection status and microbial community structure was performed using adonis function in R package vegan [65] with Bray-Curtis dissimilarity and 9999 permutations (remaining settings as default). Furthermore, association with infection load was evaluated with infection load ranging from 0 to 4 by the number of identified species within each sample. Covariates were as listed above.

### Analysis of single taxa

The relative abundance of single bacteria was evaluated from the taxonomic level of phylum to genus, and was filtered to keep most abundant taxa as follows; filtered to require a mean abundance across all samples of at least 0.05, and an abundance of 0.05 in at least one sample. Further, taxa with ≥40% zeroes across samples were removed. After filtering, 43 genera, 23 families, 15 orders, 11 classes and 5 phyla remained. Associations between the selected taxa and parasite infection status were evaluated using a linear regression with square root transformed taxa abundance and the covariates as listed above.

### Additional R-packages used

reshape2 v1.4[67], grid v3.5, gridExtra v2.3[68], gridBase v0.4[69], plyr v1.8[70], ggplot2 v3.1[71], extrafont v0.17[72], metafor v2.0[73], plotly v4.9[74], data.table v1.12[75], ggrepel v0.8[76], MASS v7.3[77], robust v0.4[64].

## Supporting information

S1 Fig Compositional alterations by increasing storage time. The illustration depicts mean cumulative abundance of taxa on **(A)** phylum, **(B)** family and **(C)** genera level for the seven different time periods for room temperature storage (each spanning 100 days). **(A)** A relative decrease in Bacteroidetes and a corresponding increase in Firmicutes is observed. The decrease in Bacteroidetes appear to be driven by Prevotellaceae at the family level **(B)**, and by *Prevotella* at the genus level **(C)**.
(PDF)

S2 Fig Taxa abundance alterations by long-term storage at room temperature. The illustration shows seven correlation plots **(A-G)** between relative abundance of selected taxa (y-axes) and storage time on filter paper in days (x-axes) with best fitted line in blue (lowess line). Most pronounced effects are seen in the Firmicutes phylum. **(H)** Beeswarm plot showing the relationship between storage time (x-axis, as seven groups each spanning 100 days) and phylodiversity (made with beeswarm function in R package beeswarm), demonstrating a significant association. Above each plot are results of a Spearman correlation analysis.
(PDF)

S3 Fig Flow-diagram for inclusion and fecal sampling. The dataset includes microscopic investigation for intestinal parasites from 1,274 children aged 2–15 years from urban Bissau, Guinea-Bissau. Details on the cohort including microscopy method is described elsewhere. A total of 1,253 samples underwent 16S rRNA gene sequencing, and 49 were excluded subsequently, yielding a final study size of 1,204 samples.
(PDF)

S1 Text Storage time associates with small but significant differences in microbiota composition. (DOCX)

S1 Table All taxa associated with intestinal parasitic infection. The table shows coefficients (Beta), significance levels (P) and Benjamini-Hochberg adjusted p-values (P.adj) for each tested association with linear regression, P.adj<0.05 across the taxonomic levels of phylum to genera.
(DOCX)
