# Supplementary material for: Intestinal protozoan infections shape fecal bacterial microbiota in children from Guinea-Bissau
Source: PLoS Negl Trop Dis. 2021 Mar 3;15(3):e0009232. doi: 10.1371/journal.pntd.0009232 (PMC7959362; doi:10.1371/journal.pntd.0009232)
Supplement: S1 Fig — The illustration depicts mean cumulative abundance of taxa on (A) phylum, (B) family and (C) genera level for the seven different time periods for room temperature storage (each spanning 100 days). (A) A relative decrease in Bacteroidetes and a corresponding increase in Firmicutes is observed. The decrease in Bacteroidetes appear to be driven by Prevotellaceae at the family level (B), and by Prevotella at the genus level (C). (PDF) [file pntd.0009232.s001.pdf]

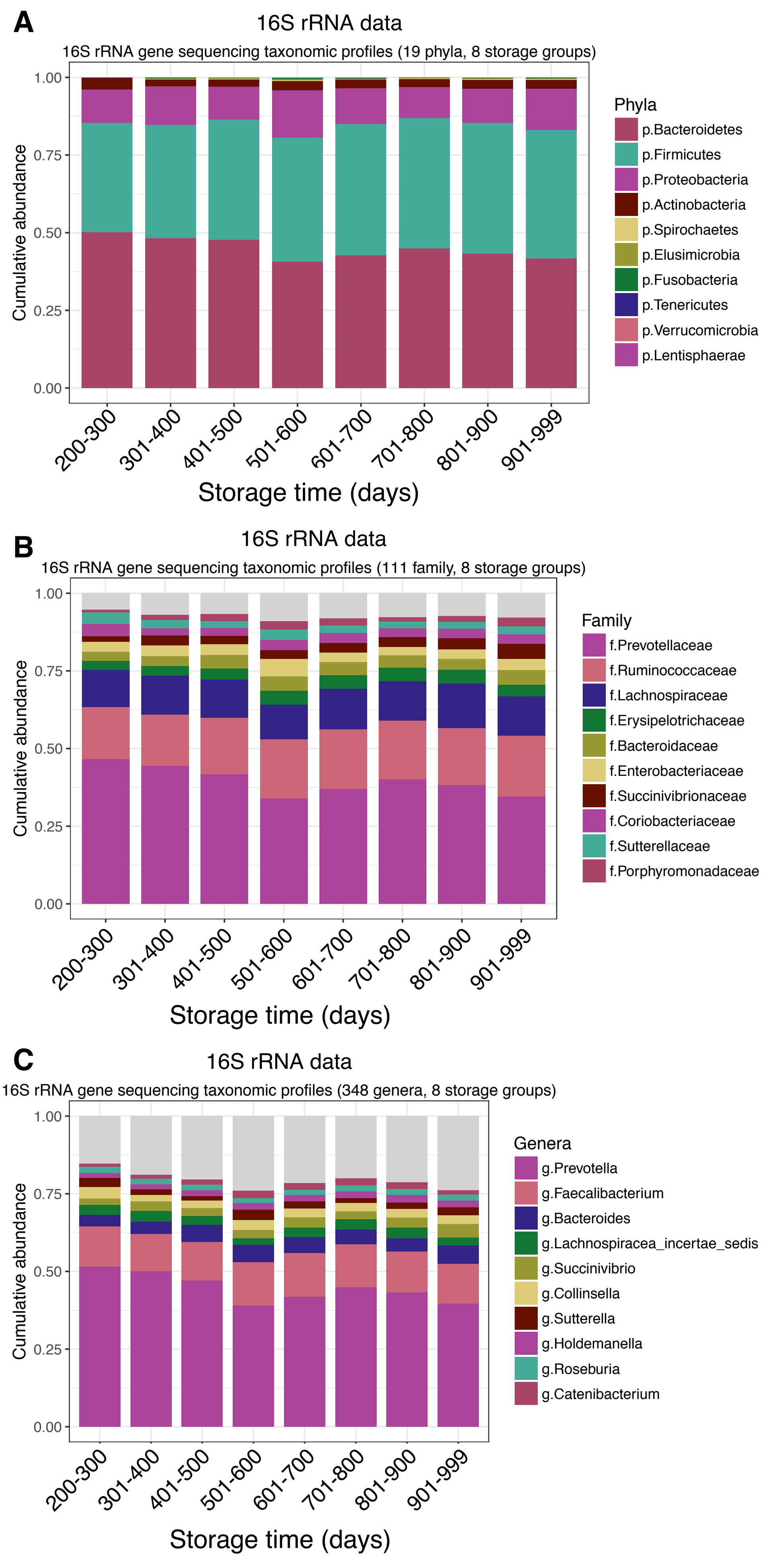

A
16S rRNA data
16S rRNA gene sequencing taxonomic profiles (19 phyla, 8 storage groups)
Cumulative abundance
1.00
0.75
0.50
0.25
0.00
200-300
301-400
401-500
501-600
601-700
701-800
801-900
901-999
Storage time (days)
Phyla
p.Bacteroidetes
p.Firmicutes
p.Proteobacteria
p.Actinobacteria
p.Spirochaetes
p.Elusimicrobia
p.Fusobacteria
p.Tenericutes
p.Verrucomicrobia
p.Lentisphaerae
B
16S rRNA data
16S rRNA gene sequencing taxonomic profiles (111 family, 8 storage groups)
Cumulative abundance
1.00
0.75
0.50
0.25
0.00
200-300
301-400
401-500
501-600
601-700
701-800
801-900
901-999
Storage time (days)
Family
f.Prevotellaceae
f.Ruminococcaceae
f.Lachnospiraceae
f.Erysipelotrichaceae
f.Bacteroidaceae
f.Enterobacteriaceae
f.Succinivibrionaceae
f.Coriobacteriaceae
f.Sutterellaceae
f.Porphyromonadaceae
C
16S rRNA data
16S rRNA gene sequencing taxonomic profiles (348 genera, 8 storage groups)
Cumulative abundance
1.00
0.75
0.50
0.25
0.00
200-300
301-400
401-500
501-600
601-700
701-800
801-900
901-999
Storage time (days)
Genera
g.Prevotella
g.Faecalibacterium
g.Bacteroides
g.Lachnospiracea_incertae_sedis
g.Succinivibrio
g.Collinsella
g.Sutterella
g.Holdemanella
g.Roseburia
g.Catenibacterium
