# Supplementary figures and images for: Intestinal protozoan infections shape fecal bacterial microbiota in children from Guinea-Bissau
Source: PLoS Negl Trop Dis. 2021 Mar 3;15(3):e0009232. doi: 10.1371/journal.pntd.0009232 (PMC7959362; doi:10.1371/journal.pntd.0009232)

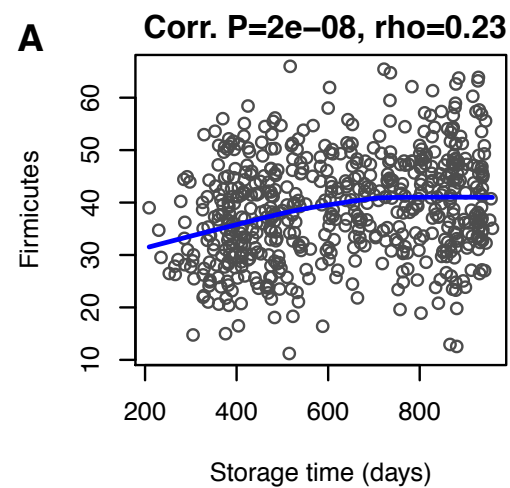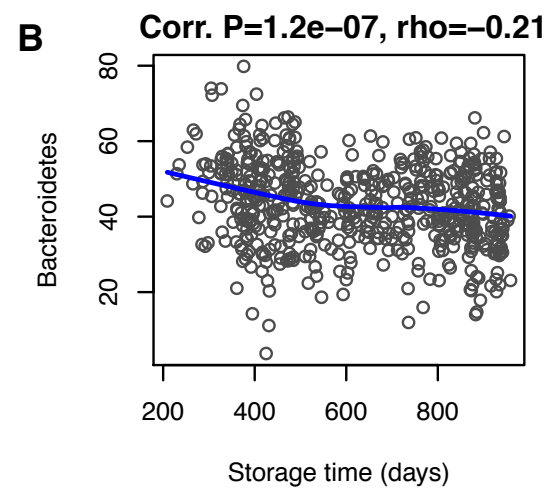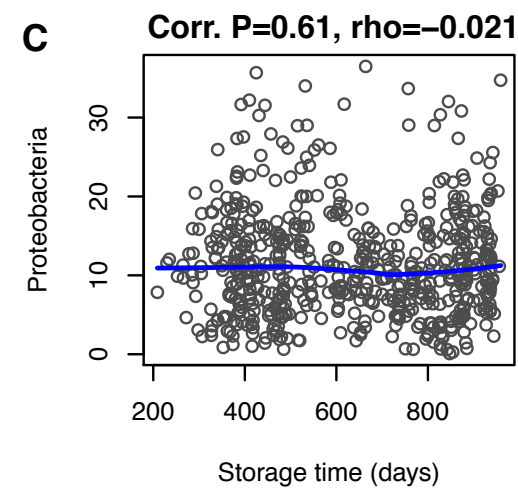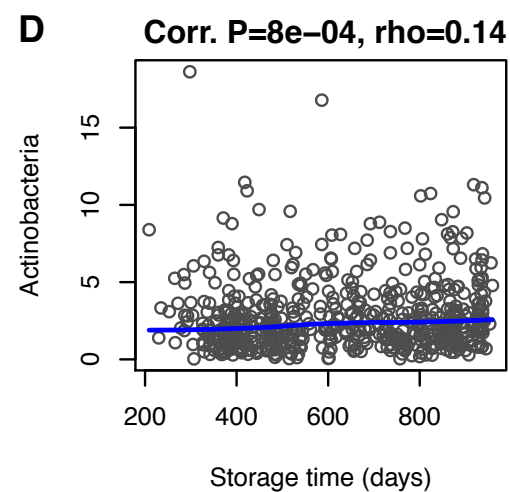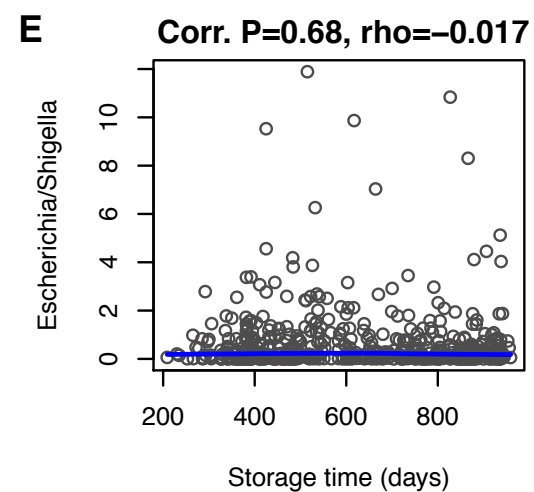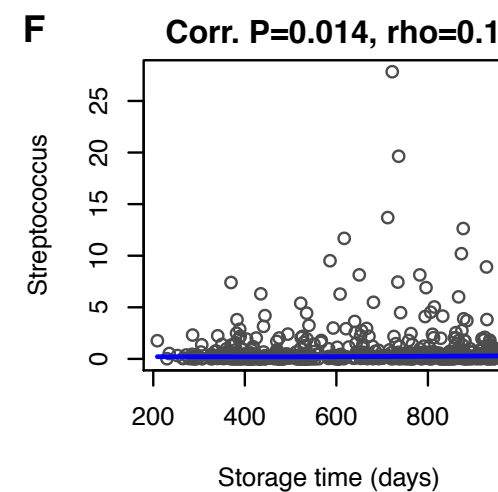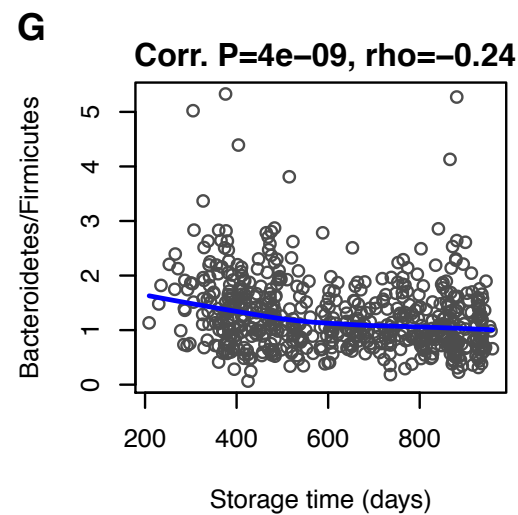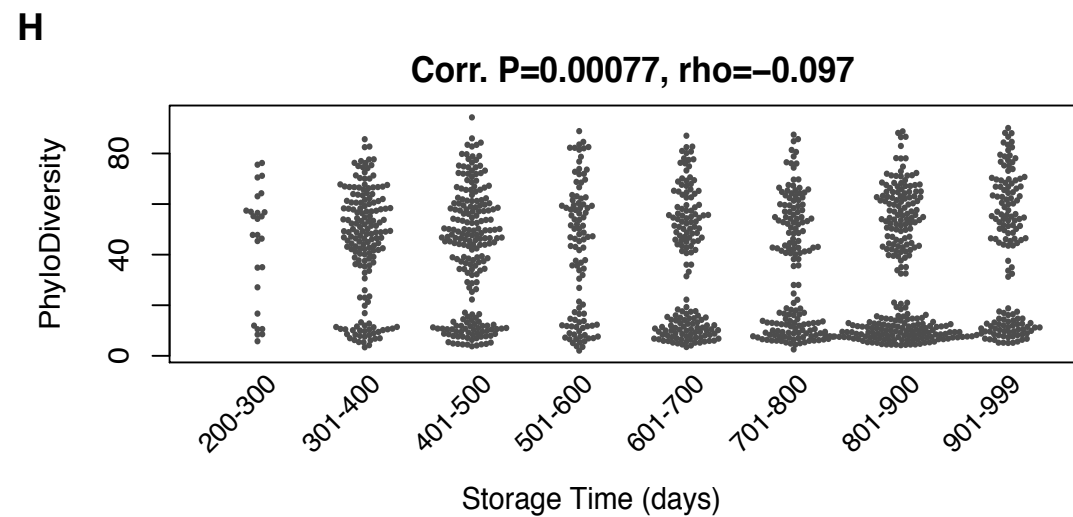

Supplement: S2 Fig — The illustration shows seven correlation plots (A-G) between relative abundance of selected taxa (y-axes) and storage time on filter paper in days (x-axes) with best fitted line in blue (lowess line). Most pronounced effects are seen in the Firmicutes phylum. (H) Beeswarm plot showing the relationship between storage time (x-axis, as seven groups each spanning 100 days) and phylodiversity (made with beeswarm function in R package beeswarm), demonstrating a significant association. Above each plot are results of a Spearman correlation analysis. (PDF) [file pntd.0009232.s002.pdf]

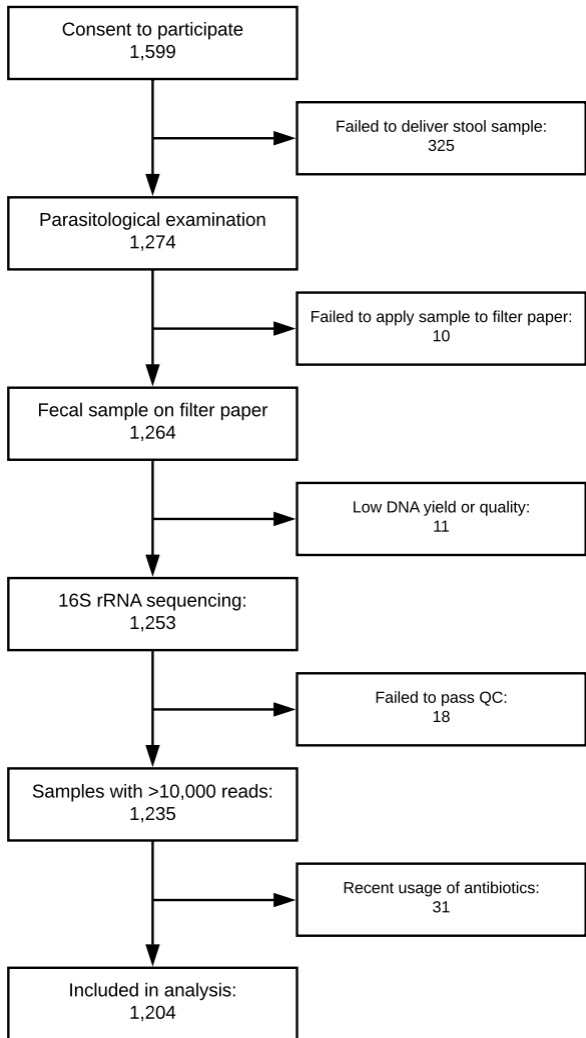

Supplement: S3 Fig — The dataset includes microscopic investigation for intestinal parasites from 1,274 children aged 2–15 years from urban Bissau, Guinea-Bissau. Details on the cohort including microscopy method is described elsewhere. A total of 1,253 samples underwent 16S rRNA gene sequencing, and 49 were excluded subsequently, yielding a final study size of 1,204 samples. (PDF) [file pntd.0009232.s003.pdf]
