# Supplementary material for: Intestinal protozoan infections shape fecal bacterial microbiota in children from Guinea-Bissau
Source: PLoS Negl Trop Dis. 2021 Mar 3;15(3):e0009232. doi: 10.1371/journal.pntd.0009232 (PMC7959362; doi:10.1371/journal.pntd.0009232)
Supplement: S1 Text — (DOCX) [file pntd.0009232.s004.docx]

**Supporting text**

*Storage time associates with small but significant differences in microbiota composition*

Due to challenges associated with collecting fecal samples in Guinea-Bissau, including unstable electricity supply and limited infrastructure to secure adequate storage at freezing temperatures, fecal samples were collected on FOBT paper and stored at ambient temperature for a longer time period. Existing studies of this procedure has reported a general good performance of the FOBT papers [1-5]. We have recently demonstrated that the storage method used in this study provide microbiota results that are very similar to conventionally stored samples kept at -80°C [6]. Within the present study, both overall DNA and sequencing quality from FOBT paper samples was satisfactory, and microbiota data resembled that of a microbiota from a conventionally stored sample. However, to limit potential confounding effects of storage time, days of storage was included as a covariate in the analyses.

We explored changes in phylogenetic diversity, beta diversity and compositional alterations of selected bacterial taxa with increasing storage time. We observed a relative increase in Bacteroidetes abundance and a corresponding decrease in Firmicutes with increasing storage time (fig. S1A). The decrease in Bacteroidetes seemed to be driven by a decreased abundance of *Prevotellaceae* at family level (Fig. S1B), and by *Prevotella* at genus level (Fig. S1C). Alpha and beta diversity associated with storage time (Spearman rho -0.097, P=0.00077, and adonis R^2^=0.048, P=0.007, respectively). Furthermore, correlation analysis of the relative abundance of selected taxa demonstrated a general significant association with storage time, however with limited average change in abundance (strongest coefficient (rho) = 0.23 observed in the Firmicutes phylum, P-values ranging from 4.0⋅10^-9^ to 6.80⋅10^-1^ for the investigated taxa) (Fig. S2).

**References**

1. Dominianni C, Wu J, Hayes RB, Ahn J. Comparison of methods for fecal microbiome biospecimen collection. BMC Microbiol. 2014;14: 103. doi:10.1186/1471-2180-14-103

2. Sinha R, Chen J, Amir A, Vogtmann E, Shi J, Inman KS, et al. Collecting Fecal Samples for Microbiome Analyses in Epidemiology Studies. Cancer Epidemiol Biomarkers Prev. 2016;25: 407–416. doi:10.1158/1055-9965.EPI-15-0951

3. Hale VL, Tan CL, Knight R, Amato KR. Effect of preservation method on spider monkey (Ateles geoffroyi) fecal microbiota over 8 weeks. J Microbiol Meth. 2015;113: 16–26. doi:10.1016/j.mimet.2015.03.021

4. Vogtmann E, Chen J, Amir A, Shi J, Abnet CC, Nelson H, et al. Comparison of Collection Methods for Fecal Samples in Microbiome Studies. Am J Epidemiol. 2017;185: 115–123. doi:10.1093/aje/kww177

5. Vogtmann E, Chen J, Kibriya MG, Chen Y, Islam T, Eunes M, et al. Comparison of Fecal Collection Methods for Microbiota Studies in Bangladesh. Appl Environ Microbiol. 2017;83. doi:10.1128/AEM.00361-17

6. Huth von S, Thingholm LB, Bang C, Rühlemann MC, Franke A, Holmskov U. Minor compositional alterations in faecal microbiota after five weeks and five months storage at room temperature on filter papers. Sci Rep. 2019;9: 19008. doi:10.1038/s41598-019-55469-0
