# Supplementary material for: Intestinal protozoan infections shape fecal bacterial microbiota in children from Guinea-Bissau
Source: PLoS Negl Trop Dis. 2021 Mar 3;15(3):e0009232. doi: 10.1371/journal.pntd.0009232 (PMC7959362; doi:10.1371/journal.pntd.0009232)
Supplement: S1 Table — The table shows coefficients (Beta), significance levels (P) and Benjamini-Hochberg adjusted p-values (P.adj) for each tested association with linear regression, P.adj<0.05 across the taxonomic levels of phylum to genera. (DOCX) [file pntd.0009232.s005.docx]

**Supplementary table 1: Taxa associated with intestinal parasitic infection.**

| **Parasite** | **Level** | **Taxa** | **All samples** | | | **Cohort II** | | **Cohort I** | |
| --- | --- | --- | --- | --- | --- | --- | --- | --- | --- |
|  |  |  | **Beta** | **P** | **P.adj** | **Beta** | **P** | **Beta** | **P** |
| Parasite pos. (overall) | Phyla | p.Actinobacteria | -1,35E-01 | 7,56E-05 | 1,58E-03 | -1,38E-01 | 3,02E-03 | -1,37E-01 | 6,37E-03 |
|  | Phyla | p.Fusobacteria | -5,18E-02 | 1,57E-03 | 1,01E-02 | -2,72E-02 | 1,46E-01 | -8,30E-02 | 3,97E-03 |
|  | Class | c.Actinobacteria | -1,35E-01 | 7,56E-05 | 7,48E-04 | -1,38E-01 | 3,02E-03 | -1,37E-01 | 6,37E-03 |
|  | Class | c.Bacilli | -1,65E-01 | 1,74E-05 | 3,44E-04 | -8,25E-02 | 6,87E-02 | -2,83E-01 | 1,59E-05 |
|  | Class | c.Betaproteobacteria | -1,75E-01 | 9,96E-05 | 8,68E-04 | -1,96E-01 | 7,97E-04 | -1,66E-01 | 1,86E-02 |
|  | Class | c.Epsilonproteobacteria | 1,43E-01 | 1,04E-06 | 6,26E-05 | 1,37E-01 | 2,44E-04 | 1,50E-01 | 1,42E-03 |
|  | Class | c.Fusobacteriia | -5,18E-02 | 1,57E-03 | 7,41E-03 | -2,72E-02 | 1,46E-01 | -8,30E-02 | 3,97E-03 |
|  | Class | c.Negativicutes | -8,47E-02 | 2,50E-03 | 1,03E-02 | -5,62E-02 | 1,06E-01 | -1,17E-01 | 1,12E-02 |
|  | Order | o.Aeromonadales | 2,01E-01 | 3,59E-03 | 1,21E-02 | 1,85E-01 | 5,98E-02 | 2,22E-01 | 2,04E-02 |
|  | Order | o.Burkholderiales | -1,62E-01 | 4,02E-04 | 2,59E-03 | -1,60E-01 | 7,48E-03 | -1,78E-01 | 1,28E-02 |
|  | Order | o.Campylobacterales | 1,43E-01 | 1,04E-06 | 2,33E-05 | 1,37E-01 | 2,44E-04 | 1,50E-01 | 1,42E-03 |
|  | Order | o.Coriobacteriales | -1,29E-01 | 1,50E-04 | 1,45E-03 | -1,37E-01 | 3,17E-03 | -1,24E-01 | 1,33E-02 |
|  | Order | o.Enterobacteriales | -2,06E-01 | 5,77E-04 | 3,24E-03 | -1,73E-01 | 1,44E-02 | -2,76E-01 | 7,07E-03 |
|  | Order | o.Fusobacteriales | -5,18E-02 | 1,57E-03 | 6,85E-03 | -2,72E-02 | 1,46E-01 | -8,30E-02 | 3,97E-03 |
|  | Order | o.Lactobacillales | -1,98E-01 | 4,59E-07 | 1,76E-05 | -1,09E-01 | 1,97E-02 | -3,24E-01 | 1,26E-06 |
|  | Order | o.Pasteurellales | -1,04E-01 | 3,40E-04 | 2,41E-03 | -9,85E-02 | 1,30E-02 | -1,18E-01 | 5,67E-03 |
|  | Order | o.Selenomonadales | -8,47E-02 | 2,50E-03 | 9,39E-03 | -5,62E-02 | 1,06E-01 | -1,17E-01 | 1,12E-02 |
|  | Family | f.Acidaminococcaceae | 8,69E-02 | 1,56E-03 | 6,09E-03 | 9,65E-02 | 3,84E-03 | 7,25E-02 | 1,16E-01 |
|  | Family | f.Bacteroidaceae | -3,81E-01 | 6,50E-08 | 1,35E-05 | -3,23E-01 | 4,93E-04 | -4,68E-01 | 2,00E-05 |
|  | Family | f.Campylobacteraceae | 1,45E-01 | 5,98E-07 | 2,06E-05 | 1,53E-01 | 3,79E-05 | 1,37E-01 | 3,40E-03 |
|  | Family | f.Catabacteriaceae | 4,58E-02 | 4,37E-04 | 2,57E-03 | 5,68E-02 | 1,03E-03 | 3,22E-02 | 1,07E-01 |
|  | Family | f.Coriobacteriaceae | -1,29E-01 | 1,50E-04 | 1,24E-03 | -1,37E-01 | 3,17E-03 | -1,24E-01 | 1,33E-02 |
|  | Family | f.Enterobacteriaceae | -2,06E-01 | 5,77E-04 | 3,23E-03 | -1,73E-01 | 1,44E-02 | -2,76E-01 | 7,07E-03 |
|  | Family | f.Eubacteriaceae | 4,64E-02 | 1,01E-03 | 4,58E-03 | 5,19E-02 | 6,51E-03 | 3,74E-02 | 7,81E-02 |
|  | Family | f.Lactobacillaceae | -1,02E-01 | 7,43E-05 | 7,33E-04 | -6,27E-02 | 4,21E-02 | -1,47E-01 | 6,74E-04 |
|  | Family | f.Pasteurellaceae | -1,04E-01 | 3,40E-04 | 2,13E-03 | -9,85E-02 | 1,30E-02 | -1,18E-01 | 5,67E-03 |
|  | Family | f.Peptostreptococcaceae | -5,22E-02 | 4,46E-04 | 2,57E-03 | -4,10E-02 | 4,00E-02 | -6,17E-02 | 5,67E-03 |
|  | Family | f.Prevotellaceae | 2,71E-01 | 1,21E-03 | 5,33E-03 | 1,87E-01 | 1,03E-01 | 4,11E-01 | 8,55E-04 |
|  | Family | f.Rikenellaceae | -7,53E-02 | 1,90E-02 | 4,98E-02 | -5,71E-02 | 1,58E-01 | -1,01E-01 | 5,27E-02 |
|  | Family | f.Streptococcaceae | -1,40E-01 | 1,54E-05 | 1,67E-04 | -7,53E-02 | 5,41E-02 | -2,36E-01 | 1,33E-05 |
|  | Family | f.Succinivibrionaceae | 2,10E-01 | 2,53E-03 | 8,73E-03 | 1,94E-01 | 5,02E-02 | 2,30E-01 | 1,67E-02 |
|  | Family | f.Sutterellaceae | -2,73E-01 | 3,59E-07 | 1,49E-05 | -2,48E-01 | 4,88E-04 | -3,14E-01 | 1,47E-04 |
|  | Family | f.Veillonellaceae | -1,76E-01 | 2,85E-06 | 7,38E-05 | -1,40E-01 | 4,62E-03 | -2,15E-01 | 2,08E-04 |
|  | Genera | g.Anaerostipes | -3,55E-02 | 1,23E-02 | 4,53E-02 | -3,63E-02 | 4,66E-02 | -3,38E-02 | 1,34E-01 |
|  | Genera | g.Bacteroides | -3,81E-01 | 6,50E-08 | 2,52E-05 | -3,23E-01 | 4,93E-04 | -4,68E-01 | 2,00E-05 |
|  | Genera | g.Campylobacter | 1,45E-01 | 6,97E-07 | 6,74E-05 | 1,53E-01 | 3,79E-05 | 1,35E-01 | 3,85E-03 |
|  | Genera | g.Catabacter | 4,58E-02 | 4,37E-04 | 3,52E-03 | 5,68E-02 | 1,03E-03 | 3,22E-02 | 1,07E-01 |
|  | Genera | g.Clostridium_IV | 4,44E-02 | 8,49E-03 | 3,35E-02 | 2,24E-02 | 2,50E-01 | 6,23E-02 | 3,21E-02 |
|  | Genera | g.Clostridium_XlVb | 2,99E-02 | 1,30E-03 | 7,74E-03 | 3,46E-02 | 1,19E-03 | 2,09E-02 | 1,97E-01 |
|  | Genera | g.Clostridium_XVIII | -1,14E-01 | 2,92E-05 | 4,71E-04 | -5,78E-02 | 1,06E-01 | -1,89E-01 | 7,82E-06 |
|  | Genera | g.Collinsella | -1,53E-01 | 8,45E-06 | 2,66E-04 | -1,55E-01 | 8,51E-04 | -1,53E-01 | 3,02E-03 |
|  | Genera | g.Escherichia.Shigella | -9,57E-02 | 8,98E-04 | 6,01E-03 | -5,64E-02 | 8,32E-02 | -1,59E-01 | 1,80E-03 |
|  | Genera | g.Eubacterium | 4,65E-02 | 9,91E-04 | 6,29E-03 | 5,19E-02 | 6,51E-03 | 3,77E-02 | 7,64E-02 |
|  | Genera | g.Haemophilus | -1,01E-01 | 2,65E-04 | 2,42E-03 | -9,49E-02 | 1,34E-02 | -1,15E-01 | 4,27E-03 |
|  | Genera | g.Lactobacillus | -1,01E-01 | 7,57E-05 | 1,01E-03 | -6,25E-02 | 4,27E-02 | -1,47E-01 | 6,74E-04 |
|  | Genera | g.Odoribacter | -5,16E-02 | 8,82E-03 | 3,45E-02 | -4,12E-02 | 7,43E-02 | -6,85E-02 | 4,48E-02 |
|  | Genera | g.Phascolarctobacterium | 9,15E-02 | 9,10E-04 | 6,01E-03 | 1,02E-01 | 2,28E-03 | 7,50E-02 | 1,04E-01 |
|  | Genera | g.Prevotella | 2,57E-01 | 1,78E-03 | 9,57E-03 | 1,84E-01 | 1,03E-01 | 3,84E-01 | 1,52E-03 |
|  | Genera | g.Roseburia | -9,79E-02 | 2,13E-03 | 1,11E-02 | -1,08E-01 | 1,25E-02 | -8,62E-02 | 7,09E-02 |
|  | Genera | g.Ruminococcus2 | -4,67E-02 | 1,21E-04 | 1,45E-03 | -2,10E-02 | 1,70E-01 | -7,98E-02 | 5,16E-05 |
|  | Genera | g.Streptococcus | -1,41E-01 | 1,32E-05 | 2,87E-04 | -7,57E-02 | 5,27E-02 | -2,37E-01 | 1,16E-05 |
|  | Genera | g.Sutterella | -2,04E-01 | 1,16E-04 | 1,45E-03 | -1,87E-01 | 6,43E-03 | -2,38E-01 | 4,18E-03 |
|  | Genera | g.Turicibacter | -4,07E-02 | 3,53E-04 | 2,91E-03 | -2,73E-02 | 5,15E-02 | -5,72E-02 | 2,53E-03 |
|  | Genera | g.Veillonella | -5,32E-02 | 8,56E-05 | 1,10E-03 | -2,32E-02 | 1,44E-01 | -9,01E-02 | 9,69E-05 |
| Helminth | Class | c.Epsilonproteobacteria | 1,34E-01 | 1,72E-02 | 4,99E-02 | 2,08E-01 | 8,58E-03 | 8,62E-02 | 2,94E-01 |
|  | Order | o.Campylobacterales | 1,34E-01 | 1,72E-02 | 4,63E-02 | 2,08E-01 | 8,58E-03 | 8,62E-02 | 2,94E-01 |
|  | Family | f.Campylobacteraceae | 1,35E-01 | 1,59E-02 | 4,27E-02 | 2,11E-01 | 6,80E-03 | 8,77E-02 | 2,88E-01 |
| Protozoa | Phyla | p.Actinobacteria | -1,42E-01 | 1,05E-04 | 1,58E-03 | -1,40E-01 | 4,83E-03 | -1,54E-01 | 4,63E-03 |
|  | Phyla | p.Fusobacteria | -6,81E-02 | 5,44E-05 | 1,58E-03 | -4,10E-02 | 3,41E-02 | -1,04E-01 | 5,26E-04 |
|  | Class | c.Actinobacteria | -1,42E-01 | 1,05E-04 | 8,68E-04 | -1,40E-01 | 4,83E-03 | -1,54E-01 | 4,63E-03 |
|  | Class | c.Bacilli | -1,71E-01 | 2,73E-05 | 4,51E-04 | -8,30E-02 | 8,55E-02 | -2,99E-01 | 1,70E-05 |
|  | Class | c.Betaproteobacteria | -1,56E-01 | 1,07E-03 | 6,22E-03 | -1,88E-01 | 2,12E-03 | -1,30E-01 | 8,59E-02 |
|  | Class | c.Epsilonproteobacteria | 1,35E-01 | 1,53E-05 | 3,44E-04 | 1,32E-01 | 7,45E-04 | 1,37E-01 | 7,69E-03 |
|  | Class | c.Erysipelotrichia | -1,10E-01 | 1,34E-02 | 4,14E-02 | -1,55E-01 | 4,49E-03 | -5,70E-02 | 4,47E-01 |
|  | Class | c.Fusobacteriia | -6,81E-02 | 5,44E-05 | 5,98E-04 | -4,10E-02 | 3,41E-02 | -1,04E-01 | 5,26E-04 |
|  | Class | c.Negativicutes | -8,87E-02 | 3,00E-03 | 1,19E-02 | -6,34E-02 | 8,73E-02 | -1,22E-01 | 1,37E-02 |
|  | Order | o.Actinomycetales | -2,17E-02 | 9,57E-03 | 2,87E-02 | -1,21E-02 | 2,40E-01 | -3,82E-02 | 5,60E-03 |
|  | Order | o.Aeromonadales | 2,20E-01 | 2,50E-03 | 9,39E-03 | 2,31E-01 | 2,49E-02 | 2,05E-01 | 4,15E-02 |
|  | Order | o.Burkholderiales | -1,44E-01 | 3,01E-03 | 1,06E-02 | -1,51E-01 | 1,65E-02 | -1,48E-01 | 5,33E-02 |
|  | Order | o.Campylobacterales | 1,35E-01 | 1,53E-05 | 2,06E-04 | 1,32E-01 | 7,45E-04 | 1,37E-01 | 7,69E-03 |
|  | Order | o.Coriobacteriales | -1,36E-01 | 2,12E-04 | 1,69E-03 | -1,39E-01 | 4,96E-03 | -1,40E-01 | 1,05E-02 |
|  | Order | o.Enterobacteriales | -2,93E-01 | 3,53E-06 | 6,80E-05 | -2,45E-01 | 8,75E-04 | -3,81E-01 | 5,21E-04 |
|  | Order | o.Erysipelotrichales | -1,10E-01 | 1,34E-02 | 3,76E-02 | -1,55E-01 | 4,49E-03 | -5,70E-02 | 4,47E-01 |
|  | Order | o.Fusobacteriales | -6,81E-02 | 5,44E-05 | 6,67E-04 | -4,10E-02 | 3,41E-02 | -1,04E-01 | 5,26E-04 |
|  | Order | o.Lactobacillales | -2,10E-01 | 4,78E-07 | 1,76E-05 | -1,11E-01 | 2,47E-02 | -3,54E-01 | 6,72E-07 |
|  | Order | o.Pasteurellales | -1,00E-01 | 1,44E-03 | 6,50E-03 | -9,48E-02 | 2,47E-02 | -1,17E-01 | 1,24E-02 |
|  | Order | o.Selenomonadales | -8,87E-02 | 3,00E-03 | 1,06E-02 | -6,34E-02 | 8,73E-02 | -1,22E-01 | 1,37E-02 |
|  | Family | f.Acidaminococcaceae | 7,47E-02 | 9,89E-03 | 2,97E-02 | 6,97E-02 | 4,88E-02 | 7,88E-02 | 1,06E-01 |
|  | Family | f.Bacteroidaceae | -3,89E-01 | 3,20E-07 | 1,49E-05 | -3,48E-01 | 4,26E-04 | -4,48E-01 | 1,97E-04 |
|  | Family | f.Campylobacteraceae | 1,39E-01 | 8,70E-06 | 1,29E-04 | 1,51E-01 | 1,05E-04 | 1,22E-01 | 1,79E-02 |
|  | Family | f.Catabacteriaceae | 4,46E-02 | 1,37E-03 | 5,79E-03 | 5,02E-02 | 5,62E-03 | 3,82E-02 | 8,09E-02 |
|  | Family | f.Coriobacteriaceae | -1,36E-01 | 2,12E-04 | 1,59E-03 | -1,39E-01 | 4,96E-03 | -1,40E-01 | 1,05E-02 |
|  | Family | f.Enterobacteriaceae | -2,93E-01 | 3,53E-06 | 7,80E-05 | -2,45E-01 | 8,75E-04 | -3,81E-01 | 5,21E-04 |
|  | Family | f.Erysipelotrichaceae | -1,10E-01 | 1,34E-02 | 3,80E-02 | -1,55E-01 | 4,49E-03 | -5,70E-02 | 4,47E-01 |
|  | Family | f.Eubacteriaceae | 4,80E-02 | 1,45E-03 | 5,88E-03 | 5,06E-02 | 1,24E-02 | 4,31E-02 | 5,88E-02 |
|  | Family | f.Lactobacillaceae | -9,97E-02 | 2,64E-04 | 1,82E-03 | -5,16E-02 | 1,19E-01 | -1,60E-01 | 5,44E-04 |
|  | Family | f.Pasteurellaceae | -1,00E-01 | 1,44E-03 | 5,88E-03 | -9,48E-02 | 2,47E-02 | -1,17E-01 | 1,24E-02 |
|  | Family | f.Peptostreptococcaceae | -5,33E-02 | 8,35E-04 | 4,02E-03 | -3,54E-02 | 9,46E-02 | -7,19E-02 | 3,09E-03 |
|  | Family | f.Prevotellaceae | 2,57E-01 | 4,45E-03 | 1,49E-02 | 1,59E-01 | 1,93E-01 | 4,13E-01 | 2,31E-03 |
|  | Family | f.Streptococcaceae | -1,49E-01 | 1,40E-05 | 1,62E-04 | -7,95E-02 | 5,65E-02 | -2,57E-01 | 7,84E-06 |
|  | Family | f.Succinivibrionaceae | 2,29E-01 | 1,78E-03 | 6,81E-03 | 2,40E-01 | 2,07E-02 | 2,14E-01 | 3,45E-02 |
|  | Family | f.Sutterellaceae | -2,83E-01 | 7,49E-07 | 2,22E-05 | -2,62E-01 | 5,05E-04 | -3,22E-01 | 3,00E-04 |
|  | Family | f.Veillonellaceae | -1,75E-01 | 1,36E-05 | 1,62E-04 | -1,37E-01 | 9,49E-03 | -2,24E-01 | 3,34E-04 |
|  | Genera | g.Bacteroides | -3,89E-01 | 3,20E-07 | 4,12E-05 | -3,48E-01 | 4,26E-04 | -4,48E-01 | 1,97E-04 |
|  | Genera | g.Butyricimonas | 5,03E-02 | 1,15E-02 | 4,28E-02 | 5,60E-02 | 3,01E-02 | 4,26E-02 | 1,75E-01 |
|  | Genera | g.Campylobacter | 1,38E-01 | 1,03E-05 | 2,66E-04 | 1,51E-01 | 1,05E-04 | 1,20E-01 | 2,02E-02 |
|  | Genera | g.Catabacter | 4,46E-02 | 1,37E-03 | 8,04E-03 | 5,02E-02 | 5,62E-03 | 3,82E-02 | 8,09E-02 |
|  | Genera | g.Clostridium_IV | 5,32E-02 | 3,13E-03 | 1,51E-02 | 2,56E-02 | 2,18E-01 | 8,35E-02 | 7,49E-03 |
|  | Genera | g.Clostridium_XlVb | 2,90E-02 | 3,29E-03 | 1,55E-02 | 3,26E-02 | 3,70E-03 | 2,28E-02 | 1,93E-01 |
|  | Genera | g.Clostridium_XVIII | -1,22E-01 | 2,91E-05 | 4,71E-04 | -6,63E-02 | 7,91E-02 | -2,02E-01 | 1,34E-05 |
|  | Genera | g.Collinsella | -1,63E-01 | 9,48E-06 | 2,66E-04 | -1,61E-01 | 1,11E-03 | -1,71E-01 | 2,30E-03 |
|  | Genera | g.Enterococcus | -3,31E-02 | 8,97E-03 | 3,45E-02 | -2,29E-02 | 1,70E-01 | -4,76E-02 | 1,55E-02 |
|  | Genera | g.Escherichia.Shigella | -1,24E-01 | 5,02E-05 | 7,20E-04 | -8,91E-02 | 7,61E-03 | -1,81E-01 | 1,13E-03 |
|  | Genera | g.Eubacterium | 4,82E-02 | 1,40E-03 | 8,10E-03 | 5,06E-02 | 1,24E-02 | 4,35E-02 | 5,66E-02 |
|  | Genera | g.Faecalibacterium | -1,32E-01 | 6,06E-03 | 2,66E-02 | -6,79E-02 | 2,38E-01 | -2,13E-01 | 8,89E-03 |
|  | Genera | g.Haemophilus | -9,47E-02 | 1,57E-03 | 8,67E-03 | -9,02E-02 | 2,72E-02 | -1,09E-01 | 1,27E-02 |
|  | Genera | g.Klebsiella | -6,19E-02 | 3,45E-03 | 1,61E-02 | -8,08E-02 | 5,09E-03 | -4,17E-02 | 1,83E-01 |
|  | Genera | g.Lactobacillus | -9,95E-02 | 2,69E-04 | 2,42E-03 | -5,14E-02 | 1,20E-01 | -1,60E-01 | 5,44E-04 |
|  | Genera | g.Odoribacter | -5,32E-02 | 1,15E-02 | 4,28E-02 | -3,51E-02 | 1,52E-01 | -7,77E-02 | 3,57E-02 |
|  | Genera | g.Oscillibacter | 6,34E-02 | 1,14E-02 | 4,28E-02 | 2,24E-02 | 4,88E-01 | 1,22E-01 | 2,12E-03 |
|  | Genera | g.Phascolarctobacterium | 7,92E-02 | 6,44E-03 | 2,77E-02 | 7,54E-02 | 3,39E-02 | 8,13E-02 | 9,59E-02 |
|  | Genera | g.Prevotella | 2,40E-01 | 7,03E-03 | 2,93E-02 | 1,53E-01 | 2,03E-01 | 3,79E-01 | 4,41E-03 |
|  | Genera | g.Roseburia | -1,11E-01 | 1,19E-03 | 7,32E-03 | -1,19E-01 | 9,49E-03 | -1,01E-01 | 5,26E-02 |
|  | Genera | g.Ruminococcus2 | -5,35E-02 | 4,03E-05 | 6,00E-04 | -2,34E-02 | 1,45E-01 | -9,33E-02 | 1,60E-05 |
|  | Genera | g.Streptococcus | -1,48E-01 | 1,54E-05 | 2,97E-04 | -7,97E-02 | 5,57E-02 | -2,54E-01 | 9,37E-06 |
|  | Genera | g.Succinivibrio | 1,77E-01 | 7,33E-03 | 3,02E-02 | 1,83E-01 | 5,27E-02 | 1,64E-01 | 6,70E-02 |
|  | Genera | g.Sutterella | -2,10E-01 | 1,79E-04 | 1,92E-03 | -1,78E-01 | 1,39E-02 | -2,61E-01 | 3,13E-03 |
|  | Genera | g.Turicibacter | -3,90E-02 | 1,48E-03 | 8,32E-03 | -2,43E-02 | 9,91E-02 | -5,80E-02 | 5,70E-03 |
|  | Genera | g.Veillonella | -5,49E-02 | 1,95E-04 | 1,98E-03 | -2,46E-02 | 1,48E-01 | -9,46E-02 | 2,24E-04 |
| Ancylostoma duodenale | Class | c.Epsilonproteobacteria | 1,64E-01 | 9,26E-03 | 3,16E-02 | 2,17E-01 | 1,64E-02 | 1,28E-01 | 1,58E-01 |
|  | Order | o.Campylobacterales | 1,64E-01 | 9,26E-03 | 2,84E-02 | 2,17E-01 | 1,64E-02 | 1,28E-01 | 1,58E-01 |
|  | Family | f.Campylobacteraceae | 1,69E-01 | 7,03E-03 | 2,24E-02 | 2,30E-01 | 9,70E-03 | 1,30E-01 | 1,55E-01 |
|  | Genera | g.Campylobacter | 1,69E-01 | 7,04E-03 | 2,93E-02 | 2,30E-01 | 9,70E-03 | 1,29E-01 | 1,56E-01 |
| Hym. nana | Family | f.Sutterellaceae | -6,74E-01 | 1,49E-02 | 4,06E-02 | -6,42E-01 | 1,34E-01 | -7,12E-01 | 5,31E-02 |
|  | Genera | g.Collinsella | -4,87E-01 | 7,99E-03 | 3,22E-02 | -2,74E-01 | 3,24E-01 | -6,84E-01 | 6,02E-03 |
| Entamoeba coli | Class | c.Bacilli | -3,25E-01 | 3,95E-05 | 5,33E-04 | -2,79E-01 | 2,08E-03 | -3,82E-01 | 4,42E-03 |
|  | Class | c.Deltaproteobacteria | 6,51E-02 | 1,07E-02 | 3,52E-02 | 8,94E-02 | 7,35E-03 | 3,43E-02 | 3,78E-01 |
|  | Class | c.Epsilonproteobacteria | 1,73E-01 | 5,68E-04 | 3,75E-03 | 2,39E-01 | 3,16E-04 | 1,04E-01 | 1,76E-01 |
|  | Class | c.Negativicutes | -1,45E-01 | 1,22E-02 | 3,91E-02 | -1,17E-01 | 1,28E-01 | -1,75E-01 | 4,84E-02 |
|  | Order | o.Actinomycetales | -4,95E-02 | 1,10E-03 | 5,30E-03 | -4,98E-02 | 5,12E-03 | -5,40E-02 | 3,27E-02 |
|  | Order | o.Aeromonadales | 6,45E-01 | 5,20E-07 | 1,76E-05 | 7,71E-01 | 8,16E-05 | 5,34E-01 | 1,09E-03 |
|  | Order | o.Campylobacterales | 1,73E-01 | 5,68E-04 | 3,24E-03 | 2,39E-01 | 3,16E-04 | 1,04E-01 | 1,76E-01 |
|  | Order | o.Desulfovibrionales | 6,55E-02 | 9,93E-03 | 2,91E-02 | 9,03E-02 | 6,41E-03 | 3,42E-02 | 3,78E-01 |
|  | Order | o.Enterobacteriales | -3,34E-01 | 5,52E-03 | 1,77E-02 | -3,05E-01 | 4,04E-02 | -4,00E-01 | 3,99E-02 |
|  | Order | o.Lactobacillales | -4,00E-01 | 7,29E-07 | 1,97E-05 | -3,50E-01 | 1,65E-04 | -4,62E-01 | 7,42E-04 |
|  | Order | o.Pasteurellales | -2,00E-01 | 7,01E-04 | 3,78E-03 | -2,74E-01 | 7,86E-04 | -1,43E-01 | 9,18E-02 |
|  | Order | o.Selenomonadales | -1,45E-01 | 1,22E-02 | 3,51E-02 | -1,17E-01 | 1,28E-01 | -1,75E-01 | 4,84E-02 |
|  | Family | f.Bacteroidaceae | -4,62E-01 | 2,10E-03 | 7,63E-03 | -3,27E-01 | 1,21E-01 | -6,05E-01 | 5,27E-03 |
|  | Family | f.Campylobacteraceae | 1,68E-01 | 7,38E-04 | 3,82E-03 | 2,55E-01 | 9,69E-05 | 8,06E-02 | 2,93E-01 |
|  | Family | f.Catabacteriaceae | 8,39E-02 | 1,02E-03 | 4,58E-03 | 8,07E-02 | 1,97E-02 | 8,86E-02 | 1,92E-02 |
|  | Family | f.Desulfovibrionaceae | 6,48E-02 | 1,05E-02 | 3,10E-02 | 8,89E-02 | 7,12E-03 | 3,43E-02 | 3,75E-01 |
|  | Family | f.Enterobacteriaceae | -3,34E-01 | 5,52E-03 | 1,81E-02 | -3,05E-01 | 4,04E-02 | -4,00E-01 | 3,99E-02 |
|  | Family | f.Lactobacillaceae | -1,75E-01 | 1,24E-03 | 5,33E-03 | -1,34E-01 | 2,54E-02 | -2,10E-01 | 2,57E-02 |
|  | Family | f.Pasteurellaceae | -2,00E-01 | 7,01E-04 | 3,72E-03 | -2,74E-01 | 7,86E-04 | -1,43E-01 | 9,18E-02 |
|  | Family | f.Streptococcaceae | -3,04E-01 | 3,79E-06 | 7,80E-05 | -2,88E-01 | 2,96E-04 | -3,35E-01 | 1,89E-03 |
|  | Family | f.Succinivibrionaceae | 6,60E-01 | 3,32E-07 | 1,49E-05 | 7,86E-01 | 6,89E-05 | 5,51E-01 | 7,88E-04 |
|  | Family | f.Sutterellaceae | -2,73E-01 | 1,14E-02 | 3,33E-02 | -3,65E-01 | 1,64E-02 | -2,09E-01 | 1,75E-01 |
|  | Family | f.Veillonellaceae | -2,61E-01 | 6,96E-04 | 3,72E-03 | -2,44E-01 | 2,14E-02 | -2,78E-01 | 1,39E-02 |
|  | Genera | g.Bacteroides | -4,62E-01 | 2,10E-03 | 1,11E-02 | -3,27E-01 | 1,21E-01 | -6,05E-01 | 5,27E-03 |
|  | Genera | g.Blautia | -1,01E-01 | 9,01E-03 | 3,45E-02 | -7,72E-02 | 1,19E-01 | -1,27E-01 | 3,81E-02 |
|  | Genera | g.Campylobacter | 1,68E-01 | 7,27E-04 | 5,52E-03 | 2,55E-01 | 9,69E-05 | 8,11E-02 | 2,90E-01 |
|  | Genera | g.Catabacter | 8,39E-02 | 1,02E-03 | 6,36E-03 | 8,07E-02 | 1,97E-02 | 8,86E-02 | 1,92E-02 |
|  | Genera | g.Catenibacterium | 2,50E-01 | 6,99E-03 | 2,93E-02 | 7,56E-02 | 5,43E-01 | 4,26E-01 | 2,02E-03 |
|  | Genera | g.Clostridium_XVIII | -2,07E-01 | 1,88E-04 | 1,96E-03 | -1,62E-01 | 3,39E-02 | -2,62E-01 | 1,34E-03 |
|  | Genera | g.Faecalibacterium | -4,02E-01 | 1,28E-05 | 2,87E-04 | -3,08E-01 | 9,18E-03 | -5,15E-01 | 3,19E-04 |
|  | Genera | g.Haemophilus | -1,91E-01 | 6,32E-04 | 4,89E-03 | -2,66E-01 | 7,02E-04 | -1,31E-01 | 9,88E-02 |
|  | Genera | g.Lachnospiracea_incertae_sedis | -2,45E-01 | 1,73E-03 | 9,43E-03 | -2,83E-01 | 1,63E-02 | -2,04E-01 | 4,08E-02 |
|  | Genera | g.Lactobacillus | -1,75E-01 | 1,24E-03 | 7,52E-03 | -1,34E-01 | 2,56E-02 | -2,10E-01 | 2,57E-02 |
|  | Genera | g.Oscillibacter | 1,32E-01 | 5,22E-03 | 2,35E-02 | 9,55E-02 | 1,52E-01 | 1,79E-01 | 7,63E-03 |
|  | Genera | g.Ruminococcus2 | -1,05E-01 | 2,04E-05 | 3,76E-04 | -6,06E-02 | 5,32E-02 | -1,48E-01 | 1,47E-04 |
|  | Genera | g.Streptococcus | -3,05E-01 | 3,53E-06 | 1,52E-04 | -2,91E-01 | 2,50E-04 | -3,32E-01 | 2,04E-03 |
|  | Genera | g.Succinivibrio | 5,85E-01 | 1,26E-06 | 8,10E-05 | 5,93E-01 | 1,19E-03 | 5,86E-01 | 1,68E-04 |
|  | Genera | g.Veillonella | -9,43E-02 | 8,90E-04 | 6,01E-03 | -9,20E-02 | 3,46E-03 | -9,67E-02 | 4,62E-02 |
| Entamoeba histolytica/dispar | Phyla | p.Actinobacteria | -1,59E-01 | 1,49E-03 | 1,01E-02 | -1,56E-01 | 2,11E-02 | -1,68E-01 | 2,54E-02 |
|  | Phyla | p.Fusobacteria | -7,67E-02 | 1,36E-03 | 1,01E-02 | -5,86E-02 | 2,32E-02 | -1,01E-01 | 2,18E-02 |
|  | Class | c.Actinobacteria | -1,59E-01 | 1,49E-03 | 7,38E-03 | -1,56E-01 | 2,11E-02 | -1,68E-01 | 2,54E-02 |
|  | Class | c.Bacilli | -2,77E-01 | 1,26E-06 | 6,26E-05 | -2,15E-01 | 5,85E-04 | -3,58E-01 | 5,21E-04 |
|  | Class | c.Epsilonproteobacteria | 1,48E-01 | 1,76E-04 | 1,34E-03 | 1,45E-01 | 2,46E-03 | 1,47E-01 | 2,64E-02 |
|  | Class | c.Erysipelotrichia | -1,92E-01 | 1,90E-03 | 8,56E-03 | -2,66E-01 | 4,55E-04 | -9,80E-02 | 3,43E-01 |
|  | Class | c.Fusobacteriia | -7,67E-02 | 1,36E-03 | 7,08E-03 | -5,86E-02 | 2,32E-02 | -1,01E-01 | 2,18E-02 |
|  | Class | c.Negativicutes | -1,44E-01 | 7,52E-04 | 4,66E-03 | -1,05E-01 | 5,00E-02 | -1,87E-01 | 7,44E-03 |
|  | Order | o.Actinomycetales | -3,86E-02 | 4,94E-04 | 3,03E-03 | -2,29E-02 | 6,25E-02 | -6,31E-02 | 1,40E-03 |
|  | Order | o.Aeromonadales | 4,43E-01 | 6,36E-06 | 1,07E-04 | 5,48E-01 | 1,34E-04 | 3,13E-01 | 1,51E-02 |
|  | Order | o.Campylobacterales | 1,48E-01 | 1,76E-04 | 1,58E-03 | 1,45E-01 | 2,46E-03 | 1,47E-01 | 2,64E-02 |
|  | Order | o.Coriobacteriales | -1,49E-01 | 3,06E-03 | 1,06E-02 | -1,52E-01 | 2,44E-02 | -1,48E-01 | 4,98E-02 |
|  | Order | o.Enterobacteriales | -3,34E-01 | 1,29E-04 | 1,34E-03 | -2,42E-01 | 1,68E-02 | -4,86E-01 | 1,47E-03 |
|  | Order | o.Erysipelotrichales | -1,92E-01 | 1,90E-03 | 8,02E-03 | -2,66E-01 | 4,55E-04 | -9,80E-02 | 3,43E-01 |
|  | Order | o.Fusobacteriales | -7,67E-02 | 1,36E-03 | 6,33E-03 | -5,86E-02 | 2,32E-02 | -1,01E-01 | 2,18E-02 |
|  | Order | o.Lactobacillales | -3,33E-01 | 1,20E-08 | 1,62E-06 | -2,57E-01 | 6,32E-05 | -4,33E-01 | 3,98E-05 |
|  | Order | o.Pasteurellales | -1,43E-01 | 7,94E-04 | 3,97E-03 | -1,18E-01 | 3,75E-02 | -1,79E-01 | 5,65E-03 |
|  | Order | o.Selenomonadales | -1,44E-01 | 7,52E-04 | 3,91E-03 | -1,05E-01 | 5,00E-02 | -1,87E-01 | 7,44E-03 |
|  | Family | f.Bacteroidaceae | -3,37E-01 | 2,34E-03 | 8,36E-03 | -3,09E-01 | 3,51E-02 | -3,83E-01 | 2,60E-02 |
|  | Family | f.Campylobacteraceae | 1,53E-01 | 9,78E-05 | 9,20E-04 | 1,65E-01 | 4,98E-04 | 1,35E-01 | 4,14E-02 |
|  | Family | f.Catabacteriaceae | 6,28E-02 | 7,79E-04 | 3,91E-03 | 7,46E-02 | 2,07E-03 | 4,67E-02 | 1,12E-01 |
|  | Family | f.Coriobacteriaceae | -1,49E-01 | 3,06E-03 | 1,04E-02 | -1,52E-01 | 2,44E-02 | -1,48E-01 | 4,98E-02 |
|  | Family | f.Enterobacteriaceae | -3,34E-01 | 1,29E-04 | 1,12E-03 | -2,42E-01 | 1,68E-02 | -4,86E-01 | 1,47E-03 |
|  | Family | f.Erysipelotrichaceae | -1,92E-01 | 1,90E-03 | 7,16E-03 | -2,66E-01 | 4,55E-04 | -9,80E-02 | 3,43E-01 |
|  | Family | f.Lachnospiraceae | -1,49E-01 | 1,87E-02 | 4,95E-02 | -1,55E-01 | 4,38E-02 | -1,41E-01 | 1,85E-01 |
|  | Family | f.Lactobacillaceae | -1,44E-01 | 1,73E-04 | 1,38E-03 | -1,20E-01 | 3,42E-03 | -1,65E-01 | 2,00E-02 |
|  | Family | f.Pasteurellaceae | -1,43E-01 | 7,94E-04 | 3,91E-03 | -1,18E-01 | 3,75E-02 | -1,79E-01 | 5,65E-03 |
|  | Family | f.Streptococcaceae | -2,52E-01 | 1,61E-07 | 1,49E-05 | -1,96E-01 | 3,95E-04 | -3,33E-01 | 7,76E-05 |
|  | Family | f.Succinivibrionaceae | 4,55E-01 | 4,15E-06 | 7,80E-05 | 5,60E-01 | 1,05E-04 | 3,24E-01 | 1,24E-02 |
|  | Family | f.Sutterellaceae | -2,91E-01 | 2,73E-04 | 1,82E-03 | -3,36E-01 | 1,54E-03 | -2,46E-01 | 4,63E-02 |
|  | Family | f.Veillonellaceae | -2,36E-01 | 3,24E-05 | 3,35E-04 | -1,93E-01 | 9,48E-03 | -2,81E-01 | 1,47E-03 |
|  | Genera | g.Bacteroides | -3,37E-01 | 2,34E-03 | 1,18E-02 | -3,09E-01 | 3,51E-02 | -3,83E-01 | 2,60E-02 |
|  | Genera | g.Blautia | -7,01E-02 | 1,36E-02 | 4,90E-02 | -5,91E-02 | 8,33E-02 | -8,78E-02 | 7,07E-02 |
|  | Genera | g.Butyricimonas | 6,65E-02 | 1,37E-02 | 4,90E-02 | 7,15E-02 | 3,83E-02 | 6,08E-02 | 1,60E-01 |
|  | Genera | g.Campylobacter | 1,49E-01 | 1,39E-04 | 1,58E-03 | 1,65E-01 | 4,98E-04 | 1,28E-01 | 5,38E-02 |
|  | Genera | g.Catabacter | 6,28E-02 | 7,79E-04 | 5,80E-03 | 7,46E-02 | 2,07E-03 | 4,67E-02 | 1,12E-01 |
|  | Genera | g.Clostridium_IV | 1,14E-01 | 7,93E-06 | 2,66E-04 | 7,07E-02 | 1,73E-02 | 1,61E-01 | 2,35E-04 |
|  | Genera | g.Clostridium_XVIII | -1,98E-01 | 9,00E-07 | 6,97E-05 | -1,80E-01 | 4,37E-04 | -2,25E-01 | 5,35E-04 |
|  | Genera | g.Collinsella | -1,82E-01 | 3,44E-04 | 2,91E-03 | -1,83E-01 | 6,57E-03 | -1,75E-01 | 2,42E-02 |
|  | Genera | g.Escherichia.Shigella | -1,53E-01 | 3,19E-04 | 2,81E-03 | -1,04E-01 | 2,19E-02 | -2,33E-01 | 2,82E-03 |
|  | Genera | g.Faecalibacterium | -2,82E-01 | 2,36E-05 | 4,15E-04 | -2,40E-01 | 3,11E-03 | -3,44E-01 | 2,07E-03 |
|  | Genera | g.Haemophilus | -1,36E-01 | 8,33E-04 | 6,00E-03 | -1,13E-01 | 3,95E-02 | -1,67E-01 | 5,76E-03 |
|  | Genera | g.Holdemanella | -1,55E-01 | 3,05E-03 | 1,49E-02 | -1,20E-01 | 6,19E-02 | -1,80E-01 | 3,96E-02 |
|  | Genera | g.Lachnospiracea_incertae_sedis | -1,64E-01 | 5,27E-03 | 2,35E-02 | -1,75E-01 | 3,36E-02 | -1,29E-01 | 1,13E-01 |
|  | Genera | g.Lactobacillus | -1,44E-01 | 1,75E-04 | 1,92E-03 | -1,19E-01 | 3,45E-03 | -1,65E-01 | 2,00E-02 |
|  | Genera | g.Oscillibacter | 1,08E-01 | 2,16E-03 | 1,11E-02 | 4,94E-02 | 2,80E-01 | 1,89E-01 | 6,34E-04 |
|  | Genera | g.Roseburia | -1,27E-01 | 8,00E-03 | 3,22E-02 | -1,24E-01 | 5,44E-02 | -1,31E-01 | 7,24E-02 |
|  | Genera | g.Ruminococcus2 | -8,43E-02 | 2,84E-06 | 1,48E-04 | -6,30E-02 | 3,45E-03 | -1,12E-01 | 2,75E-04 |
|  | Genera | g.Streptococcus | -2,50E-01 | 2,03E-07 | 3,92E-05 | -1,94E-01 | 4,41E-04 | -3,30E-01 | 8,97E-05 |
|  | Genera | g.Succinivibrio | 3,79E-01 | 3,04E-05 | 4,71E-04 | 4,30E-01 | 1,12E-03 | 3,16E-01 | 8,98E-03 |
|  | Genera | g.Sutterella | -2,62E-01 | 8,38E-04 | 6,00E-03 | -3,05E-01 | 2,54E-03 | -2,16E-01 | 8,29E-02 |
|  | Genera | g.Veillonella | -7,40E-02 | 3,49E-04 | 2,91E-03 | -5,31E-02 | 1,33E-02 | -9,51E-02 | 1,28E-02 |
| Giardia lamblia | Phyla | p.Actinobacteria | -1,51E-01 | 1,21E-03 | 1,01E-02 | -1,36E-01 | 2,70E-02 | -1,87E-01 | 9,43E-03 |
|  | Phyla | p.Fusobacteria | -6,91E-02 | 2,24E-03 | 1,26E-02 | -4,74E-02 | 5,82E-02 | -1,02E-01 | 1,48E-02 |
|  | Class | c.Actinobacteria | -1,51E-01 | 1,21E-03 | 6,67E-03 | -1,36E-01 | 2,70E-02 | -1,87E-01 | 9,43E-03 |
|  | Class | c.Betaproteobacteria | -2,43E-01 | 4,31E-05 | 5,33E-04 | -2,68E-01 | 3,59E-04 | -2,53E-01 | 8,60E-03 |
|  | Class | c.Epsilonproteobacteria | 1,66E-01 | 1,12E-05 | 3,44E-04 | 1,45E-01 | 1,79E-03 | 1,99E-01 | 1,95E-03 |
|  | Class | c.Erysipelotrichia | -1,37E-01 | 1,46E-02 | 4,38E-02 | -1,68E-01 | 1,50E-02 | -9,47E-02 | 3,22E-01 |
|  | Class | c.Fusobacteriia | -6,91E-02 | 2,24E-03 | 9,62E-03 | -4,74E-02 | 5,82E-02 | -1,02E-01 | 1,48E-02 |
|  | Order | o.Burkholderiales | -2,35E-01 | 1,07E-04 | 1,20E-03 | -2,25E-01 | 3,94E-03 | -2,90E-01 | 2,89E-03 |
|  | Order | o.Campylobacterales | 1,66E-01 | 1,12E-05 | 1,68E-04 | 1,45E-01 | 1,79E-03 | 1,99E-01 | 1,95E-03 |
|  | Order | o.Coriobacteriales | -1,45E-01 | 1,98E-03 | 8,12E-03 | -1,33E-01 | 3,15E-02 | -1,77E-01 | 1,51E-02 |
|  | Order | o.Enterobacteriales | -2,82E-01 | 3,97E-04 | 2,59E-03 | -2,56E-01 | 4,61E-03 | -3,55E-01 | 1,36E-02 |
|  | Order | o.Erysipelotrichales | -1,37E-01 | 1,46E-02 | 4,02E-02 | -1,68E-01 | 1,50E-02 | -9,47E-02 | 3,22E-01 |
|  | Order | o.Fusobacteriales | -6,91E-02 | 2,24E-03 | 8,88E-03 | -4,74E-02 | 5,82E-02 | -1,02E-01 | 1,48E-02 |
|  | Family | f.Bacteroidaceae | -4,23E-01 | 1,41E-05 | 1,62E-04 | -4,00E-01 | 1,29E-03 | -4,50E-01 | 4,52E-03 |
|  | Family | f.Campylobacteraceae | 1,68E-01 | 7,63E-06 | 1,22E-04 | 1,66E-01 | 3,30E-04 | 1,79E-01 | 5,43E-03 |
|  | Family | f.Coriobacteriaceae | -1,45E-01 | 1,98E-03 | 7,33E-03 | -1,33E-01 | 3,15E-02 | -1,77E-01 | 1,51E-02 |
|  | Family | f.Enterobacteriaceae | -2,82E-01 | 3,97E-04 | 2,42E-03 | -2,56E-01 | 4,61E-03 | -3,55E-01 | 1,36E-02 |
|  | Family | f.Erysipelotrichaceae | -1,37E-01 | 1,46E-02 | 4,03E-02 | -1,68E-01 | 1,50E-02 | -9,47E-02 | 3,22E-01 |
|  | Family | f.Eubacteriaceae | 5,99E-02 | 1,52E-03 | 6,05E-03 | 5,42E-02 | 2,92E-02 | 7,07E-02 | 1,64E-02 |
|  | Family | f.Peptostreptococcaceae | -5,50E-02 | 6,28E-03 | 2,03E-02 | -4,03E-02 | 1,20E-01 | -6,90E-02 | 3,14E-02 |
|  | Family | f.Prevotellaceae | 4,14E-01 | 3,35E-04 | 2,13E-03 | 3,59E-01 | 1,90E-02 | 5,36E-01 | 2,57E-03 |
|  | Family | f.Rikenellaceae | -1,09E-01 | 1,39E-02 | 3,90E-02 | -8,98E-02 | 8,84E-02 | -1,40E-01 | 6,99E-02 |
|  | Family | f.Sutterellaceae | -3,19E-01 | 6,93E-06 | 1,20E-04 | -2,61E-01 | 4,85E-03 | -4,17E-01 | 1,95E-04 |
|  | Family | f.Veillonellaceae | -1,34E-01 | 8,82E-03 | 2,68E-02 | -1,17E-01 | 7,35E-02 | -1,72E-01 | 4,09E-02 |
|  | Genera | g.Bacteroides | -4,23E-01 | 1,41E-05 | 2,87E-04 | -4,00E-01 | 1,29E-03 | -4,50E-01 | 4,52E-03 |
|  | Genera | g.Campylobacter | 1,66E-01 | 1,01E-05 | 2,66E-04 | 1,66E-01 | 3,30E-04 | 1,74E-01 | 6,89E-03 |
|  | Genera | g.Collinsella | -1,63E-01 | 5,47E-04 | 4,32E-03 | -1,36E-01 | 2,78E-02 | -2,14E-01 | 4,03E-03 |
|  | Genera | g.Enterococcus | -4,37E-02 | 6,30E-03 | 2,74E-02 | -3,28E-02 | 1,02E-01 | -6,17E-02 | 1,98E-02 |
|  | Genera | g.Eubacterium | 6,01E-02 | 1,48E-03 | 8,32E-03 | 5,42E-02 | 2,92E-02 | 7,11E-02 | 1,59E-02 |
|  | Genera | g.Fusicatenibacter | -5,96E-02 | 3,16E-03 | 1,51E-02 | -7,58E-02 | 2,69E-03 | -3,68E-02 | 2,74E-01 |
|  | Genera | g.Klebsiella | -7,51E-02 | 4,99E-03 | 2,27E-02 | -9,25E-02 | 8,86E-03 | -5,65E-02 | 1,74E-01 |
|  | Genera | g.Prevotella | 3,80E-01 | 8,53E-04 | 6,00E-03 | 3,41E-01 | 2,39E-02 | 4,71E-01 | 7,06E-03 |
|  | Genera | g.Sutterella | -2,32E-01 | 9,16E-04 | 6,01E-03 | -1,37E-01 | 1,29E-01 | -3,92E-01 | 5,13E-04 |
|  | Genera | g.Turicibacter | -4,25E-02 | 8,11E-03 | 3,24E-02 | -2,61E-02 | 1,63E-01 | -6,64E-02 | 2,07E-02 |
| Endolimax nana | Class | c.Bacilli | -2,31E-01 | 4,83E-03 | 1,77E-02 | -1,17E-01 | 1,96E-01 | -3,94E-01 | 6,86E-03 |
|  | Class | c.Deltaproteobacteria | 7,13E-02 | 5,97E-03 | 2,11E-02 | 9,74E-02 | 2,82E-03 | 4,44E-02 | 2,87E-01 |
|  | Class | c.Epsilonproteobacteria | 1,93E-01 | 1,90E-04 | 1,34E-03 | 1,96E-01 | 2,29E-03 | 1,94E-01 | 2,32E-02 |
|  | Class | c.Negativicutes | -1,68E-01 | 4,68E-03 | 1,77E-02 | -8,67E-02 | 2,43E-01 | -2,63E-01 | 6,68E-03 |
|  | Order | o.Campylobacterales | 1,93E-01 | 1,90E-04 | 1,60E-03 | 1,96E-01 | 2,29E-03 | 1,94E-01 | 2,32E-02 |
|  | Order | o.Desulfovibrionales | 6,85E-02 | 8,01E-03 | 2,51E-02 | 9,30E-02 | 4,10E-03 | 4,34E-02 | 2,96E-01 |
|  | Order | o.Lactobacillales | -3,01E-01 | 3,07E-04 | 2,30E-03 | -1,54E-01 | 9,57E-02 | -5,00E-01 | 7,78E-04 |
|  | Order | o.Selenomonadales | -1,68E-01 | 4,68E-03 | 1,54E-02 | -8,67E-02 | 2,43E-01 | -2,63E-01 | 6,68E-03 |
|  | Family | f.Campylobacteraceae | 1,98E-01 | 1,24E-04 | 1,12E-03 | 2,14E-01 | 6,97E-04 | 1,82E-01 | 3,40E-02 |
|  | Family | f.Catabacteriaceae | 1,22E-01 | 9,32E-06 | 1,29E-04 | 1,10E-01 | 1,87E-03 | 1,29E-01 | 2,77E-03 |
|  | Family | f.Desulfovibrionaceae | 6,83E-02 | 7,93E-03 | 2,49E-02 | 9,31E-02 | 3,92E-03 | 4,27E-02 | 3,02E-01 |
|  | Family | f.Eubacteriaceae | 1,05E-01 | 2,15E-04 | 1,59E-03 | 1,23E-01 | 1,85E-03 | 7,39E-02 | 6,84E-02 |
|  | Family | f.Lachnospiraceae | -2,65E-01 | 2,47E-03 | 8,66E-03 | -3,28E-01 | 2,16E-03 | -2,17E-01 | 1,36E-01 |
|  | Family | f.Lactobacillaceae | -1,39E-01 | 1,25E-02 | 3,58E-02 | -8,05E-02 | 1,76E-01 | -2,07E-01 | 4,25E-02 |
|  | Family | f.Streptococcaceae | -2,27E-01 | 9,28E-04 | 4,37E-03 | -9,99E-02 | 2,12E-01 | -4,03E-01 | 5,74E-04 |
|  | Family | f.Sutterellaceae | -4,00E-01 | 2,64E-04 | 1,82E-03 | -3,81E-01 | 9,49E-03 | -4,26E-01 | 1,05E-02 |
|  | Family | f.Veillonellaceae | -2,08E-01 | 8,31E-03 | 2,57E-02 | -1,98E-01 | 5,60E-02 | -2,31E-01 | 5,90E-02 |
|  | Genera | g.Campylobacter | 1,98E-01 | 1,23E-04 | 1,45E-03 | 2,14E-01 | 6,97E-04 | 1,82E-01 | 3,38E-02 |
|  | Genera | g.Catabacter | 1,22E-01 | 9,32E-06 | 2,66E-04 | 1,10E-01 | 1,87E-03 | 1,29E-01 | 2,77E-03 |
|  | Genera | g.Clostridium_IV | 1,02E-01 | 2,54E-03 | 1,26E-02 | 4,96E-02 | 2,36E-01 | 1,55E-01 | 4,20E-03 |
|  | Genera | g.Clostridium_XlVb | 8,30E-02 | 1,40E-05 | 2,87E-04 | 9,03E-02 | 4,03E-05 | 7,45E-02 | 2,50E-02 |
|  | Genera | g.Clostridium_XVIII | -2,25E-01 | 5,44E-05 | 7,52E-04 | -1,57E-01 | 3,35E-02 | -3,02E-01 | 4,55E-04 |
|  | Genera | g.Eubacterium | 1,05E-01 | 2,14E-04 | 2,13E-03 | 1,23E-01 | 1,85E-03 | 7,40E-02 | 6,82E-02 |
|  | Genera | g.Lactobacillus | -1,39E-01 | 1,25E-02 | 4,57E-02 | -8,03E-02 | 1,77E-01 | -2,07E-01 | 4,25E-02 |
|  | Genera | g.Oscillibacter | 1,82E-01 | 2,25E-04 | 2,18E-03 | 1,14E-01 | 8,39E-02 | 2,63E-01 | 4,58E-04 |
|  | Genera | g.Roseburia | -3,10E-01 | 3,06E-06 | 1,48E-04 | -3,30E-01 | 2,08E-04 | -2,92E-01 | 3,83E-03 |
|  | Genera | g.Ruminococcus2 | -7,70E-02 | 2,29E-03 | 1,16E-02 | -5,31E-02 | 8,24E-02 | -1,12E-01 | 8,16E-03 |
|  | Genera | g.Streptococcus | -2,27E-01 | 9,41E-04 | 6,07E-03 | -1,01E-01 | 2,04E-01 | -4,00E-01 | 6,36E-04 |
|  | Genera | g.Sutterella | -3,95E-01 | 2,46E-04 | 2,32E-03 | -3,94E-01 | 5,15E-03 | -4,10E-01 | 1,44E-02 |
|  | Genera | g.Veillonella | -8,27E-02 | 4,30E-03 | 1,98E-02 | -5,44E-02 | 7,35E-02 | -1,16E-01 | 2,78E-02 |
